# Multidimensional School-Based and Family-Involved Interventions to Promote a Healthy and Sustainable Lifestyle (LIVELY) for Childhood Obesity Prevention: Study Protocol

**DOI:** 10.2196/57509

**Published:** 2024-10-30

**Authors:** Sara Basilico, Maria Vittoria Conti, Ilaria Ardoino, Chiara Breda, Federica Loperfido, Elena Klaic, Linda Spialtini, Andreana Foresta, Francesca Orsini, Maria Luisa Ojeda Fernandez, Stefano Conca Bonizzoni, Elisabetta Modena, Yasamin Tootoonchi Hamedani, Federica Villa, Hellas Cena, Marta Baviera, Carlotta Franchi

**Affiliations:** 1 Laboratory of Dietetics and Clinical Nutrition Department of Public Health, Experimental and Forensic Medicine University of Pavia Pavia Italy; 2 Laboratory of Pharmacoepidemiology and Human Nutrition Department of Health Policy Istituto di Ricerche Farmacologiche Mario Negri Istituto di Ricovero e Cura a Carattere Scientifico Milan Italy; 3 Laboratory of Cardiovascular Prevention Department of Health Policy Istituto di Ricerche Farmacologiche Mario Negri Istituto di Ricovero e Cura a Carattere Scientifico Milan Italy; 4 Department of Humanities University of Pavia Pavia Italy; 5 Clinical Nutrition Unit Istituti Clinici Scientifici Maugeri Istituto di Ricovero e Cura a Carattere Scientifico Pavia Italy; 6 Italian Institute for Planetary Health Milan Italy

**Keywords:** childhood obesity, school education, family education, healthy lifestyle, sustainability, primary prevention, nutritional intervention, physical activity, community engagement, lifestyle habits, socio-economic status, LIVELY study

## Abstract

**Background:**

Childhood obesity has become a significant public health concern over the past 2 decades, posing multifactorial challenges, including modifiable factors like dietary habits, physical activity, screen time, and sleeping habits. Prevention efforts require a comprehensive approach comprising educational interventions, collaboration among multidisciplinary teams, and community engagement. Since schools play a central role in children's lives, they are the ideal setting for promoting healthy habits.

**Objective:**

The LIVELY (Multidimensional School-Based and Family-Involved Interventions to Promote a Healthy and Sustainable Lifestyle) study will assess the prevalence of overweight and obesity in primary school children and identify contributing factors within families. Additionally, it aims to implement and evaluate a multidimensional, multidisciplinary intervention to foster a sustainable and healthy lifestyle, ultimately working toward preventing obesity in school-aged children.

**Methods:**

During the school year, each class will be individually involved in a multidimensional educational intervention covering the topics of healthy, sustainable nutrition and lifestyle. Children will also participate in a multimedia lab where they will create an animated cartoon. The lectures will engage them through various methods, including direct instructions, games, and drawing activities, to stimulate and enhance their learning and involvement.

**Results:**

Data collection began in October 2023 and will last until the end of October 2024. A sample of 227 children from 14 classes was included in the study. The mean age was 8.9 (SD 1.2) years, and 48% (n=110) were males. Among the overall sample, 18.1% (n=41; 95% CI 13.7%-23.7%) were overweight, while 5.3% (n=12; 95% CI 3%-9%) had obesity. Males had a higher prevalence of obesity than females (9.1%, n=10 vs 1.7%, n=3, *P*=.03, respectively). Otherwise, the prevalence of central obesity was similar between the two (*P=.*329). Data analysis and the presentation of the complete results will be available after the end of 2024.

**Conclusions:**

The study could lead to the structuring of an educational intervention model in school settings aimed at preventing childhood obesity. Moreover, it could help raise awareness of childhood obesity and help prevent this public health issue.

**Trial Registration:**

ClinicalTrials.gov NCT05966051; https://clinicaltrials.gov/study/NCT05966051

**International Registered Report Identifier (IRRID):**

DERR1-10.2196/57509

## Introduction

### Background

Childhood overweight and obesity have emerged as public health crises, reaching a global dimension [1]. Overweight and obesity prevalence estimates from the fifth round of Childhood Obesity Surveillance Initiative (COSI) data collection (2018-2020), conducted in 33 countries of the World Health Organization (WHO) European Region, indicate that 29% of children aged 7 to 9 years are living with overweight, including 12% with obesity, with Italy ranking fourth highest [2].

Primary factors contributing to obesity in Europe—unhealthy dietary habits, lack of physical activity, high screen time, and poor sleep habits—stand as prominent drivers of preventable fatalities, chronic illnesses, and economic health burdens among young people. These modifiable determinants have been particularly challenged during the COVID-19 pandemic, across all population age groups, including children. The lockdown significantly impacted children’s lifestyle habits, contributing to increased sedentary behavior, extended screen time, and higher consumption of calorie-dense and sugary foods [3].

Total screen time, time spent on television, and time spent on the computer are positively correlated with overweight/obesity among children [4]. The pandemic has heightened the use of technology platforms, on the one hand, to facilitate communication and, on the other hand, to facilitate virtual education. Indeed, it has been reported that screen time has increased by about 5 hours per day compared to the pre–COVID-19 period [3]. However, this has exacerbated sedentary habits and increased snack consumption, resulting in weight gain [5]. Prolonged screen time can also affect the quality and quantity of sleep hours [6]. Inadequate sleep negatively influences several aspects of children’s and adolescents' lives, including physical and mental health and school performance [7]. Inadequate sleep is also correlated with increased cardiometabolic risk, leading to the onset of impaired fasting blood glucose, dyslipidemia, and increased blood pressure [8]. Along with excessive screen time, inadequate sleep is also associated with unhealthy eating behaviors. This could lead to an increased risk of obesity and overweight as evidenced by recent meta-analyses [9,10]

In such a sensitive and complex scenario, where many factors play a central role in maintaining good health conditions, more evidence-based policies, economic investments, and integrated programs are required [11,12]. In the past decades, several childhood abatement interventions have been carried out to address this issue, but increasing regional and global prevalence trends show that those have had little success [2,13,14]. Scientific evidence described how the “eat less, move more” attitude is too simplistic, and no single intervention can be successful in halting the advancement of the obesity epidemic. Therefore, modern policy actions have been prompted into executing integrated programs, which take into consideration: (1) multicomponent approaches including nutrition, physical activity, sleep hygiene, and so on; (2) multilevel efforts, including individual children, families, and school; and (3) multi-setting interventions, such as primary care clinics, homes, and community centers to prevent, contain, and manage childhood overweight and obesity [2,15,16].

Adherence to the Mediterranean diet (MD) and lifestyle represents a further piece of the puzzle as the primary prevention strategy for obesity recognized by many relevant scientific societies [17]. However, MD adoption has decreased in the past decades in most WHO member states, including countries in the Mediterranean basin and among younger individuals belonging to lower socioeconomic classes [18-20]. According to estimates of the most recent COSI data collected (2018-2020), less than half (43%) of the children in the WHO European Region consumed fresh fruit daily, and even less (34%) ate vegetables daily [2]. According to these data, their level of physical activity remains poor as well. Overall, 40% of children did not spend any time during the week doing sports, while 43% of children aged 6 to 9 years had at least 2 hours a day of screen time, with the prevalence reaching 76% on the weekends [2].

Italy is no exception. The transition toward Western dietary patterns, based on ultraprocessed foods and a sedentary lifestyle, is the consequence of numerous sociocultural and economic factors, such as an increased cost of living, changes in gender roles in society, urbanization, and globalization [21]. In particular, children of migrant backgrounds often shift away from traditional eating habits, increasing their consumption of processed foods, likely due to economic barriers [20,21]. As of January 1, 2020, Italy had 1.3 million children of migrant background aged 0 to 17 years, so it is clear that instilling nutritional education and promoting healthy lifestyle habits within this population group is crucial for reducing childhood obesity rates in the coming years [22].

Since establishing healthy dietary habits at an early age may prevent the onset of chronic diseases later in adulthood, schools have been recognized as powerful settings to provide nutritional and healthy lifestyle education and execute targeted interventions [15,23].

Archero et al [24] conducted a 2-month cross-sectional study in Novara, including 3 primary and 2 secondary schools, to assess the association between MD and weight status. The authors found a higher prevalence of overweight and obesity and a lower adherence to MD in children attending primary school compared to other schools. Another cross-sectional study was conducted by Paduano et al [25] to investigate the physical/sedentary activities of first-year primary school children in Modena and their association with overweight/obesity and dietary habits. The authors found that 3 out of 4 children spent less than 7 hours a week engaged in physical activities, while 63.9% dedicated 2 or more hours a day to sedentary activities. Recently, the Italian National Institute of Health, in collaboration with the Ministry of Health, implemented a project, called “Maestra Natura” [26] aimed at promoting healthy lifestyles and improving knowledge about food and nutrition in elementary school children. The results showed a significant improvement in knowledge (*P* <.001) in the intervention group compared to the control group.

Despite several actions carried out to counteract childhood obesity in Italy [24-26], the rising incidence of obesity demonstrates that the measures adopted have been ineffective at achieving their short- and long-term goals [27]. In this context, the LIVELY (Multidimensional School-Based and Family-Involved Interventions to Promote a Healthy and Sustainable Lifestyle) study aims to address the prevalence of overweight and obesity among primary school children and its determinants, as well as to set up and evaluate the feasibility of a multidimensional school-based educational intervention for preventing childhood obesity.

### Study Objectives

#### Primary Objective

The study’s primary objective is to investigate the prevalence of overweight and obesity among primary school children, as well as its determinants, including the children’s clinical history and lifestyle habits, and their families’ socioeconomic status, environmental influences, and behavioral factors.

#### Secondary Objectives

The secondary aims are to (1) evaluate the feasibility (in terms of satisfaction, learning, and organizational impact) of a multidimensional school-based educational intervention aimed at promoting a healthy and sustainable lifestyle to prevent childhood obesity; and (2) assess changes in children’s diet and lifestyle, as well as in family behavioral attitudes, at 6 and 12 months after the intervention.

## Methods

### Study Design and Overview

LIVELY is a single-arm pre-post study (without a control group). The study is being conducted by a multidisciplinary team consisting of researchers from the Istituto di Ricerche Farmacologiche Mario Negri IRCCS (Istituto di Ricovero e Cura a Carattere Scientifico) of Milan and the Laboratory of Dietetics and Clinical Nutrition from the University of Pavia.

### Study Setting

**Figure 1:**
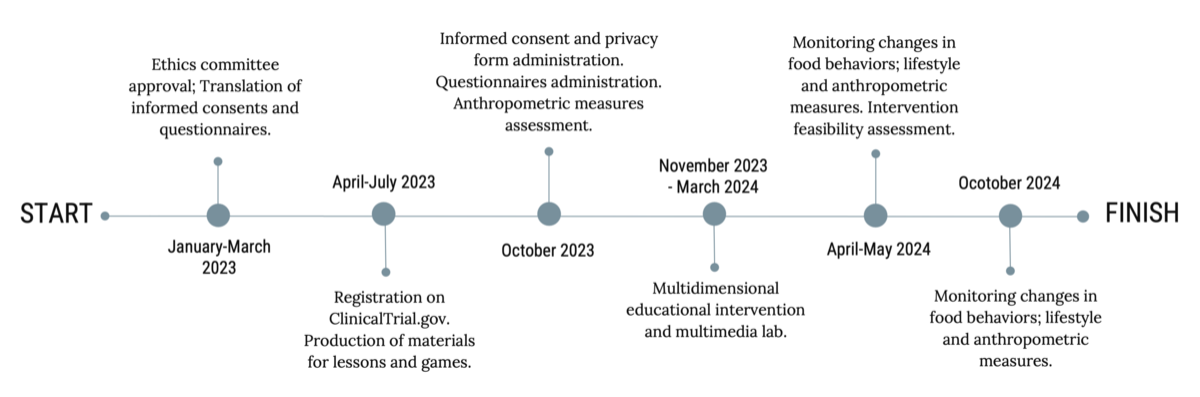
Study flowchart with timeline.

LIVELY is being carried out at Istituto Comprensivo ”Luigi Cadorna,“ a public primary school with a strong multiethnic component in northwest Milan, Italy. The study will run from October 2023 until October 2024. Figure 1 shows the study flowchart and timeline.

### Selection Criteria

The target population for the study was children from 5 to 12 years of age, of both sexes and any ethnicity, and their families. Only children whose parents or legal guardians approved and signed the informed consent were included.

### Recruitment

The multidisciplinary team organized an initial meeting at the participating school to introduce the project to the dean and teachers. Teacher representatives took charge of presenting the project to the families of the various classes. Interested families were provided with a study information sheet, which was translated into different languages (Arabic and English) to accommodate the school’s diverse, primarily Arabian, population. Teachers in participating classes distributed informed consent and privacy forms in the families’ native languages. Signed documents were required from all families before their children could participate.

### Ethical Considerations

The LIVELY study was approved by the Fondazione IRCCS Istituto Neurologico Carlo Besta Ethics Committee (protocol 11) in January 2023. It follows the principles of the Declaration of Helsinki and complies with all relevant privacy and confidentiality regulations concerning the participants' data. Written informed consent was obtained from parents or legal guardians before data collection. Each participant was assigned a unique code to link their data securely while protecting their identity.

### Outcome Measures

Details of the data collected from the children and their families throughout the study period are summarized in Table 1.

**Table 1:** Summary of outcome measures collected from the participating children and their families^a^.

Data collection points and outcomes			Baseline (T0)	6 months (T1)	12 months (T2)
**Anthropometry measurements**					
	Weight		✓	✓	✓
	Height		✓	✓	✓
	Waist circumference		✓	✓	✓
	Bicep circumference		✓	✓	✓
**Questionnaires**					
	**Sociodemographic information about the parents**				
		Household composition	✓	✓	✓
		Ethnicity	✓		
		Education	✓	✓	✓
		Job	✓	✓	✓
		Health	✓	✓	✓
		Smoking and drinking habits	✓	✓	✓
		Household food habits	✓	✓	✓
	**Sociodemographic information about the children**				
		Age	✓		
		Attended class	✓		✓
		Health conditions	✓	✓	✓
	**Children’s diet and lifestyle information**				
		Adherence to Mediterranean Diet	✓	✓	✓
		Consumption of ultra-processed foods	✓	✓	✓
		Water consumption	✓	✓	✓
		Sleep habits	✓	✓	✓
		Screen time habits	✓	✓	✓
	**Project evaluation**				
		Feasibility of the project (satisfaction, learning, and organization)		✓	

^a^The table shows all the outcome measures that will be collected at baseline (T0), 6 months (T1), and 12 months (T2) after the start of the study from the participating children and their families.

#### Anthropometric Measurements and Weight Status Classification

A team of trained nutritionists conducted the initial assessment of the children’s anthropometric measurements at T0, with plans to reassess these measurements at T1 and T2.

The children’s weight was measured using the same digital scale (Tanita Corp). Before standing on the scale, the children were asked to remove their shoes and to wear just trousers and a T-shirt. Body weight was recorded to the nearest 0.1 kilogram. Height was measured using a portable altimeter (Seca GmbH & Co) and recorded to the nearest millimeter. Waist circumference (WC) was measured midway between the lower rib and the iliac crest to the nearest 0.5 cm, while the circumference of the nondominant bicep was measured with the forearm flexed 90°, between the acromion process and the olecranon to the nearest 1 cm. 

BMI was expressed as BMI *z* score by referring to the growth curves proposed by the WHO that classify children as having underweight, normal weight, overweight (BMI by sex and age greater than 2 SDs), or obesity (BMI greater than 3 standard deviations) [28]. The software Anthro and AnthroPlus (both developed by the World Health Organization) were used to calculate BMI *z* score for the children [29].

The waist-to-height ratio (WHtR) was calculated as WC divided by height, both expressed in centimeters. For the definition of central distribution of adiposity, we adopted the discriminatory value of 0.5 for WHtR [30-32], which corresponds to the 85th percentile of our sample [33,34]. Pupils were separated into 2 groups: the first one included children with WHtR <0.5 (normal distribution of adiposity), while the second one included children with WHtR ≥0.5 (central obesity).

#### Data Collection

##### Overview

A case report form (CRF) was used to collect information regarding the children and their household and to investigate all the determinants of overweight and obesity (Multimedia Appendix 1). Referring teachers distributed the CRF in paper format to each child participating in the study in the family's native language and collected them once they were filled out by the parents.

##### Sociodemographic Information

Sociodemographic and socioeconomic information about the family (household composition, ethnicity, parents' education, job, health, smoking and drinking habits, and household food habits) and the children (age, attended class, health conditions, and practices related to child feeding) were collected through a structured questionnaire at the beginning of the school year (T0).

##### Diet and Lifestyle

Information regarding the child's eating habits and lifestyle were assessed at T0 through a structured questionnaire administered to the parent or legal guardians. The same questionnaire will be administered at 6 months (T1) and 12 months (T2) after the beginning of the study to assess changes in children's eating and lifestyle habits in relation to the educational content addressed in the classrooms during the intervention.

Specifically, the Mediterranean Diet Quality Index for Children and Adolescents (KIDMED) [35,36] questionnaire was used to assess adherence to the MD and provide an overview of the children’s consumption of major food groups (fruits, vegetables, pasta and cereals, legumes, fish, meat, and dairy products). The questionnaire also gathered details about the number of meals consumed, use of the school cafeteria, sweeteners in tea or milk (eg, sweetened cocoa, sugar, or honey), consumption of whole foods, and intake of ultraprocessed foods (NOVA classification) [37,38]. In the absence of a validated questionnaire to assess children's lifestyle, questions were structured based on the 2018 Centro di Ricerca Alimenti e Nutrizione (CREA) Guidelines [39] to assess hydration level and the WHO guidelines [40] to assess physical activity, sleep habits, and screen time.

### Multidimensional Educational Interventions

#### Lessons and Games

During the school year, each class participates in a multidimensional educational intervention focused on healthy and sustainable nutrition and lifestyle. This intervention is being led by experienced staff, including nutritional biologists and registered dietitians, and was integrated into Civic Education lessons during regular school hours. From October 2023 to March 2024, 6 lectures plus a multimedia workshop are scheduled, covering topics such as macronutrients, micronutrients, the digestive system, the food pyramid, the healthy eating plate, and lifestyle (including sleep habits, screen time, and physical activity).

Researchers visit the school 3 days a week, working with 2 classes per day: one during the first 2 hours of the morning and another in the 2 hours before lunch. Each 2-hour session takes place every 4 weeks and is divided into 2 parts: 30 to 40 minutes of direct teaching using interactive slides, followed by 80 to 90 minutes of playful activities tailored to the children's age group. Activities include crossword puzzles, hangman, and memory games focused on the topics discussed. Another proposed activity involves depicting an unmarked food pyramid and magnetic cards depicting different foods; the goal was to stick the cards at the correct pyramid level (eg, ultraprocessed foods at the top of the pyramid). The children are also asked to come up with combinations of foods to achieve a healthy, balanced plate. At the end of each lecture, to increase family involvement and reinforce learning, a summary slide of each lesson is sent to the parents. Multimedia Appendix 2 includes the teaching presentation used for the second lecture on micronutrients, along with 2 summaries provided to the parents.

#### Multimedia Workshop

After the first 5 lessons, in which children will have learned the basics of healthy and sustainable nutrition, a multimedia workshop will be conducted to create an animated cartoon. This activity, designed by the Officine Creative of the University of Pavia, aims to foster discussions about healthy eating, promote creative narrative reflection, and document the activities carried out in class.

The goal is to reinforce the content learned in previous lessons, stimulate motivation and discussion among the students, and guide them through character design and the creation of shared dramaturgies.

The children, divided into small groups, are asked to draw a humanized food item, giving it a name, distinguishing features, personality, and clothing (Multimedia Appendix 3). Based on their drawings and character descriptions, each group will then develop a dramaturgy. To help structure these stories, the children will use “story inventor” cubes, with each side of the cube displaying typical fairy tale elements, such as the setting, the helper, the magical object, or an unexpected event. Each image on the cube thus serves as a prompt for the creation of shared dramaturgies. The images and tales produced by the children will be collected, processed, and animated using augmented reality techniques, allowing the children to experience the stories they created in a dynamic, immersive way. Table 2 summarizes the lectures and activities proposed for the intervention.

**Table 2:** Summary of the lectures and activities proposed during the interventiona.

Lectures title	Covered topics	Games/activities	Lecture aim	Time period
1. Macronutrients	Carbohydrates, proteins; lipids; water	True or false; crossword puzzle; game of sets; rhyming riddles	Learn what macronutrients are, their functions, importance to our bodies, and what foods they are present in. Know the importance of water, main functions, and daily intake.	November 2023
2. Micronutrients	Vitamins and minerals	Rhyming riddles; the hangman's game; memory game	Learn what micronutrients are, their functions, their importance to our bodies, and what foods they are present in.	November to December 2023
3. Digestive system	The pathway of food from the mouth to the esophagus, to the stomach, to the intestines; the enzymes involved; and where major macronutrients are absorbed	World search; true or false; rhyming riddles; a vision of a short cartoon on the digestive system; human anatomy labeling game	Learn what happens to food when we eat, what path it takes inside our bodies, how food is digested, and how nutrients are absorbed.	December 2023
4. Food pyramid	Composition of the food pyramid; frequency of food consumption; ultraprocessed foods; food labels	Rebus; food pyramid sorting game; riddles	Learn the frequency of food consumption; know the foods to consume every day and those to consume only occasionally. Learn what ultraprocessed foods are and learn how to read food labels.	January 2024
5. Healthy eating plate	Plate composition; how to compose a healthy dish; importance of breakfast; seasonality of fruits and vegetables	Create your own healthy dish game; Find the Odd One Out game; Find the Missing Part game	Learn how to compose a balanced, healthy dish; learn about alternatives for each part of the dish (fiber, carbohydrates, and protein); learn how to compose a healthy dish on your own by choosing seasonal foods.	January to February 2024
6. Lifestyle	Sleep habits; screen time; physical activity	True or false; riddles; complete your healthy day activity	Know what good habits are to stay healthy and lead an active lifestyle; know the difference between moderate and intense physical activity and related examples; learn how much time should be devoted to these activities; know how many hours of sleep a child needs and what are good habits before sleeping; know what is the limit of screen time and what alternative activities are.	March 2024
7. Multimedia lab	Drawing and storytelling	Drawing a humanized food and proposing a dramaturgy	Work on the content learned in previous lessons, activate motivation and discussion among students; work on character design and the creation of shared dramaturgies.	February to March 2024

^a^The table presents the various lectures and activities that make up the educational intervention given during the LIVELY project. For each lecture, the topics covered, the games or activities planned, the learning objective, and the time period are described.

### Evaluation of the Project 

The evaluation of educational interventions will aim to analyze and interpret relevant aspects of the multidimensional intervention. It will involve both the school team (teachers) and the recipients (children and families) participating in the LIVELY project and will be conducted at the end of the school year (T1).

The feasibility of the multidimensional educational intervention at school will be assessed in terms of satisfaction, learning, and organization. Three main areas of evaluation of educational interventions will be distinguished, namely, satisfaction (by children, families, and teachers), learning (by children), and organizational impact in the work context (by teachers).

The children's and their families' satisfaction will be assessed through an ad hoc questionnaire investigating the level of appreciation regarding the proposed activities. The children's knowledge of the topics covered in the lessons (healthy and sustainable nutrition and lifestyle) will be collected through age-appropriate questionnaires (true/false items, multiple-choice items, insertion) or short compositions, depending on their age group. This assessment will help us determine whether the concepts explained in the lessons have been learned and whether the educational model used is appropriate for the target population. The overall satisfaction and organizational impact of the interventions will be evaluated by teachers through an ad hoc structured questionnaire, which will explore the following issues: (1) usefulness (definition and consistency/congruence of the multidimensional interventions with the project objectives, (2) usefulness of the interventions and materials prepared), (3) didactics (competence and appropriateness of the teaching techniques of the study teams addressed to the subjects, support received by the study teams during the implementation of the activities), (4) organization, and (5) services (timing, facilities and appropriateness of the schools' premises). In addition, teachers will be asked to refer to the reproducibility of the intervention in other classrooms or schools. All items in the questionnaire will be rated on a Likert-type scale.

### Sample Size Calculation

This type of study did not require a sample size calculation, but we expected to enroll about 300 to 350 children aged between 6 and 12 years from 15 to 20 classes (in primary school and in the first class of secondary school) based on the availability/willingness of teachers to participate and the resources.

This would allow us to estimate the prevalence of overweight and obesity between 23% and 24%, with a 20% relative precision and within a 95% CI. These data are in line with those observed in the Okkio alla Salute 2019 survey in the Lombardy region [41].

### Statistical Analysis

Descriptive analysis will be performed to summarize the baseline characteristics of the involved children according to normal weight, overweight, and obesity classifications. Descriptive data were expressed as counts (percentages) for categorical data and as means (SD) for continuous variables. The means for continuous variables were compared using independent group *t* tests when the data were normally distributed; othewise, the Mann-Whitney test was applied. Categorical variables were compared using the chi-square test. Main determinants of overweight/obesity among children will be investigated according to clinical conditions, dietary patterns, physical activity habits, and environmental and behavioral (family) conditions collected at the baseline using logistic/multinomial regression models.

Specifically, according to the questionnaire results and the information collected in the CRF, children will be identified as “fully adherent,” “partially adherent,” or “poorly adherent” to the WHO recommendations regarding eating and lifestyle habits [42].

The obesogenic environment will be investigated by summarizing all information collected with an appropriate index by assigning a score ranging from 0 (least obesogenic) to 100 (most obesogenic). Each item will be assigned a score from 0 to 1: variables that were considered positive aspects of the environment (grocery, stores/superstores, exercise opportunities) will be reverse scored so that a lower score for these variables indicates a healthier environment. Variables that are considered negative aspects (fast food restaurants, food budgets at home, using a car to go to school) will be scored as is, so a higher score indicates an unhealthy environment. The final index will be calculated by summing up the score for the single items and then normalized to lie between 0 and 100.

Indices for each dimension and an aggregate index will also be calculated to assess the level of satisfaction with the training intervention, learning and overall knowledge of the topic, and the intervention’s organization and reproducibility. Multivariate statistical analysis will be used to investigate general and specific quality dimensions and factors, and it will be correlated with the other aforementioned measures. Changes in dietary and lifestyle habits in the children, behavioral attitudes of their families, and the children's anthropometric measurements will be assessed at 6 (T1) and 12 months (T2) starting from baseline. All results will be stratified by sex and age groups.

## Results

### Preliminary Results

Recruitment lasted throughout October 2023. Out of the 275 children from the 14 classes participating in the project, 227 whose parents approved and signed the informed consent were included in the study.

The mean age was 8.9 (SD 1.2) years, and 48% (n=110) were males. Children from the 14 classes were distributed as follows: 5.3% (n=12 children) from the second year (1 class), 33.9% (n=77 children) from the third year (5 classes), 15.9% (n=36 children) from the fourth year (2 classes), and 44.9% (n=102 children) from the fifth year (6 classes). No first-graders took part in the project. Data related to demographic data, including age, ethnicity, weight, height, BMI *z* score, and WHtR are shown for the total population and by sex in Table 3.

**Table 3:** Demographic characteristics of the study population^a^.

Characteristics		Study population			*P* value	
		Total (n=227, 100%)	Male (n=110, 48%)	Females (n=117, 52%)		
Age (years), mean (SD)		8.9 (1.2)	8.9 (1.2)	8.9 (1.1)	0.975^b^	
Age range (years)		6-12	6-12	7-12		
**Place of birth, n (%)**						0.091^c^
	Italy	170 (79.3)	83 (75.5)	97 (82.9)		
	Europe	10 (4.4)	9 (8.2)	1 (0.9)		
	South America	11 (4.8)	6 (5.5)	5 (4.3)		
	Africa	23 (10.1)	10 (9.1)	13 (11.1)		
	Asia	3 (1.3)	2 (1.8)	1 (0.9)		
**Ethnicity, n (%)**						0.858^c^
	Caucasian	83 (36.6)	44 (40)	39 (33.3)		
	North African	80 (35.2)	35 (31.8)	45 (38.5)		
	Latin American	31 (13.7)	16 (14.5)	15 (12.8)		
	Asian	20 (8.8)	9 (8.2)	11 (9.4)		
	Black African	4 (1.8)	1 (0.9)	3 (2.6)		
	Mixed	9 (4)	5 (4.5)	4 (3.4)		
**Attended class, n (%)**						0.640^c^
	First-year class	0 (0)	0 (0)	0 (0)		
	Second-year class	12 (5.3)	8 (7.3)	4 (3.4)		
	Third-year class	77 (33.9)	36 (32.7)	41 (35)		
	Fourth-year class	36 (15.9)	17 (15.5)	19 (16.2)		
	Fifth-year class	102 (44.9)	49 (44.5)	53 (45.3)		
**Anthropometric measurements, mean (SD)**						
	Weight (kg)	35.86 (10.53)	35.27 (10.5)	36.41 (10.57)	0.419^b^	
	Height (cm)	136.33 (8.74)	135.85 (8.38)	136.75 (99)	0.420^b^	
	Waist circumference (cm)	65.19 (11.37)	65.22 (10.95)	65.15 (11.79)	0.966^b^	
	Bicep circumference (cm)	22.11 (41)	21.72 (4.12)	22.47 (3.89)	0.159^b^	
	WHtR^d^	0.48 (07)	0.48 (07)	0.48 (07)	0.666^b^	
	BMI *z* score	0.87 (1.42)	0.87 (1.55)	0.87 (1.30)	0.971^b^	
**BMI z score classes, n (%)**						0.025^c^
	Underweight	5 (2.2)	4 (3.6)	1 (0.9)		
	Normal weight	169 (74.4)	80 (72.7)	89 (76.1)		
	Overweight	41 (18.1)	16 (14.5)	24 (21.4)		
	Obesity	12 (5.3)	10 (9.1)	3 (1.7)		
**Central obesity, n (%)**						0.329^c^
	WHtR <0.5	150 (66.1)	76 (69%)	74 (63%)		
	WHtR ≥0.5	77 (33.9)	34 (31%)	43 (37%)		

^a^Table shows the demographic and anthropometric characteristics of the overall study population and then divided between male and female students. Categorical variables are presented as n (%), and continuous variables are presented as mean (SD).

^b^*P* value of the *t* test between male and female students.

^c^*P* value of the chi-square test between male and female students.

^d^WHtR: waist-to-height ratio.

The percentage of normal weight, overweight, and obesity (defined by BMI *z* score) and central obesity (according to the WHtR cut-off) is reported in Table 4. Among the overall sample, 18.1% (n=41; 95% CI 13.7%-23.7%) were overweight, while 5.3% (n=12; 95% CI 3%-9%) were obese. Male students had a higher prevalence of obesity than female students (9.1% vs 1.7%, *P*=.03 respectively). Conversely, the overall prevalence of central obesity was similar between male and female students, respectively (31% vs 37%, *P*=.329).

**Table 4:** Weight status prevalence according to BMI z score and its relation to WHtR^a^ in male and female students.

Participants				Total, n (%)		WHtR <0.5, n (%)		WHtR ≥0.5, n (%)
**Males**				110 (100)		76 (69)		34 (31)
	**BMI *z* score (WHO^b^)**							
		Underweight	4 (3.6)		4 (5.3)		0 (0)	
		Normal weight	80 (72.7)		71 (93.4)		9 (26.5)	
		Overweight	16 (14.5)		1 (1.3)		15 (44.1)	
		Obesity	10 (9.1)		0 (0)		10 (29.4)	
**Females**				117 (100)		74 (63)		43 (37)
	**BMI** ***z*** **score (WHO)**							
		Underweight	1 (0.9)		1 (1.4)		0 (0)	
		Normal weight	89 (76.1)		71 (95.9)		18 (41.9)	
		Overweight	24 (21.4)		2 (2.7)		23 (53.5)	
		Obesity	3 (1.7)		0 (0)		2 (4.7)	

^a^WHtR: waist-to-height ratio.

**^b^**WHO: World Health Organization.

The second data collection was completed in June 2024 (T1), and the last data collection will be completed by the end of October 2024 (T2). Data analysis and a presentation of the complete results will be available after the end of 2024. 

## Discussion

### Overview

The rising prevalence of obesity worldwide represents a growing burden of noncommunicable diseases (NCDs) and a significant public health issue due to increasing public service health costs [13,18,43]. To prevent the early onset of NCDs in younger populations, additional policies, treatments, and financial investments must be made to contrast the growing pandemic of childhood obesity [44-46]. Based on scientific research [15], preventing childhood obesity requires a multifaceted approach including multiple areas of intervention. Therefore, it is essential to develop and carry out integrated programs that could cover a variety of topics (nutrition, physical activity, sleep hygiene, etc) at various levels (individual, family, school, and institutional) [47].

The LIVELY study enrolled 227 school-age children attending an elementary school in a multiethnic neighborhood in north Milan, Italy. Classes from the second to the fifth grade were included. No first-grade class joined the project because the availability of children to follow the lesson could not be optimal. This was because, during the first year of elementary school, children were just beginning to learn how to read and write, leading primary school teachers to abstain from participating in the project.

In line with previous studies [24-26], our preliminary results showed a high prevalence of overweight/obesity among the enrolled children, at 24% (n=26) for the male students and 23% (n=27) for the female students, with a higher prevalence of obesity among males (*P*=.007). Interestingly, the prevalence of central obesity was similar between the 2 sexes (*P*=.329).

### Study Strengths

A child's diet and physical activity can be significantly influenced by their surroundings, particularly family and school [48]. To date, many programs have been developed to prevent obesity in children, but the overall impact of these is questionable and does not include long-term follow-up measurements [24-26]. Thus, the LIVELY study has significant strengths. First, interventions involving different actors (children, families, teachers) and multidisciplinary professional expertise will be implemented, allowing us to address the different determinants of childhood obesity. Second, frontal lectures and games relating to the subject matter will be adapted to all age groups, dividing the children into 2 groups—first and second graders in one group, and third to fifth graders in the other. The strong multicultural component of the sample will be considered by involving them in stimulating and bonding activities. Third, an interactive digital lab will be developed to maximize learning outcomes by taking advantage of the children’s digital skills. Fourth, attention will be given to the opinions of all participants (children, parents, and teachers) to assess the feasibility and reproducibility of the project.

### Study Limitations

There are several possible obstacles to this research in real-world contexts; some that we can foresee now and others that will be managed as they arise. We have already encountered 2 main challenges. The first challenge comprised the teachers’ active involvement and participation. Some teachers expressed that they found it difficult to include new lectures in the already tight teaching schedule as all classes are conducted during regular school hours and included within the Civic Education class period. However, it was possible to overcome this obstacle by involving the school principal and explaining to teachers the importance and impact of the project since the topics covered are not part of the regular school curriculum and will cover current and important issues such as sustainability and planetary health. Moreover, this approach aligns with the Sustainable Development Goals outlined in the 2030 Agenda for Sustainable Development, adopted by all United Nations Member States, and integrated into Civic Education curricula by the Italian Ministry of Education in 2020. The second challenge concerned the large multicultural component of the sample, as language barriers can play a critical role. To minimize this obstacle, most of the tools concerning completion by families (informed consent, releases, and questionnaires) were translated into 3 different languages (Italian, English, and Arabic).

We anticipate challenges with family compliance in completing the self-administered questionnaires. To address this, we plan to make phone calls to the families to fill in the questionnaires or enter the missing data with the support of trained staff. Another challenge may arise from the difficulty in collecting data at 12 months from children transitioning from 5th grade to 6th grade, as they will be changing school locations. We plan to mitigate this issue by maintaining contact with teachers and parents. In addition, through satisfaction questionnaires, the project’s various stakeholders will be given a voice to highlight the strengths and address any critical issues through different perspectives.

### Conclusions

The LIVELY study will provide key insights into the prevalence of overweight and obesity in a primary school setting and identify their main determinants. It is expected to benefit the study population by conveying, through a multidisciplinary, multiprofessional, and reproducible approach, the basics of a healthy and sustainable lifestyle. The ultimate goal is to reduce—and altogether prevent—childhood obesity.

## Appendix

Multimedia Appendix 1 Case report form.

Multimedia Appendix 2 Lectures on micronutrients.

Multimedia Appendix 3 Multimedia lab activity example.

Multimedia Appendix 4 SPIRIT Checklist.

## Abbreviations

COSI: Childhood Obesity Surveillance Initiative
CREA: Centro di Ricerca Alimenti e Nutrizione
CRF: case report form
CVD: cardiovascular disease
IRCCS: Istituto di Ricovero e Cura a Carattere Scientifico
KIDMED: Mediterranean Diet Quality Index for Children and Adolescents
LIVELY: Multidimensional School-Based and Family-Involved Interventions to Promote a Healthy and Sustainable Lifestyle
LDNC: Laboratory of Dietetics and Clinical Nutrition
MD: Mediterranean diet
NCD: noncommunicable disease
WC: waist circumference
WHO: World Health Organization
WHtR: waist-to-height ratio
UNESCO: United Nations Educational, Scientific and Cultural Organization

## Data Availability

The data that support the findings of this study are available from the corresponding author upon reasonable request, subject to prior authorization from the Istituto di Ricerche Farmacologiche Mario Negri (IRCCS) of Milan, the data controller complying with the General Data Protection Regulations (GDPR). Data sharing is restricted to comply with the regulations of the European Economic Area (EEA). As such, data sharing with entities or individuals located outside the EEA is not foreseen.
