# Supplementary material for: Multidimensional School-Based and Family-Involved Interventions to Promote a Healthy and Sustainable Lifestyle (LIVELY) for Childhood Obesity Prevention: Study Protocol
Source: JMIR Res Protoc. 2024 Oct 30;13:e57509. doi: 10.2196/57509 (PMC11561434; doi:10.2196/57509)
Supplement: Multimedia Appendix 1 [file resprot_v13i1e57509_app1.pdf]

Mu**L**tidimensional school-based and family **I**nvolved inter**V**entions, to promote a h**E**althy and sustainable **L**ifest**Y**le for the childhood obesity primary prevention: the **LIVELY** study

Dear parent,

We ask for about 10 minutes of your time to fill out this form. We will ask you some questions, which are of interest to the LIVELY Study.

Date: ____ / ____ / ______

Name and surname of the child: ____

Place of birth of the child: ____________________________

Date of birth of the child: ____ / ____ / __________

Sex: ☐ F ☐ M

School attended: ☐ Dolci ☐ King

**Grade:**	☐ II grade	**Section:**	☐ A
	☐ III grade		☐ B
	☐ IV grade		☐ C
	☐ V grade		☐ D
	☐ VI grade		☐ E

What is your relationship to the child?

☐ Mother ☐ Father ☐ Other family member: ____________

Including the participating child(ren), how many people live together in the same household?

☐ Mother ☐ Sons → state the number: __________

☐ Father ☐ Daughters → state the number: _______

☐ Grandparents → state the number ____ ☐ Others → state the number: __________

The child has been diagnosed with a food-related disorder?

□ Yes □ No

If yes, which of the following?

□ Food intolerances and/or allergies. Please, specify which: ____________________________

□ Type 1 diabetes mellitus

□ Endocrine disorders (e.g., dysthyroidism, hypercorticism) ____________________________

□ Celiac disease

□ Other metabolic disorders (phenylketonuria, dyslipidemia, etc.).

Please, specify which: ____

**PERCEPTIONS ABOUT THE CHILD**		
In your opinion, the child is eating:		
□ Too little	□ The right amount	□ Too much food
In your opinion, the child is:		
□ Underweight	□ A little overweight	
□ Normalweight	□ Very overweight	
In your opinion, the amount of time the child spend on physical activity (movement games, sports, etc.) is?		
□ Just a bit	□ Enough	□ A lot

Is any household member currently overweight or affected by obesity?

☐ Yes ☐ No

If yes, please specify who: ____

Was any adult household member overweight or affected by obesity at a young age?

☐ Yes ☐ No

If yes, please specify who: ____

Is there anyone in your family diagnosed with Diabetes Mellitus type 1 or 2?

☐ Yes ☐ No

If yes, please specify who: ____

Do you have shared times in your family when you engage in physical activity? (e.g. walking, cycling, going to the park, etc.)

☐ Yes ☐ No

Mother's data

Age: ______ Country of origin: ___________________________

Weight: ______kg Height: _______ cm

Education degree:

☐ None ☐ Elementary school diploma ☐ Middle school diploma

☐ High school diploma ☐ University degree (BSc/MSc/other)

Job: ____

Smoking status:

☐ Current smoker ☐ Nover smoked ☐ Former smoker

Alcohol consumption:

☐ No consumption

☐ Wine, beer, aperitifs:

The consumption is:

□ Occasional consumption

□ Habitual consumption → n° of glasses per day: ____

☐ Liquor and/or spirits:

The consumption is:

□ Occasional consumption

□ Habitual consumption → n° of glasses per day: ____

Do you exercise regularly?

☐ Yes ☐ No

How do you usually spend your free time? (*watching TV, reading, using smartphone, playing videogames, walking, gardening, dog walking, cycling, etc…*)

____

____

____

____

FATHER’S DATA

Age: ______ Country of origin: ___________________________

Weight: ______kg Height: _______ cm

Education degree:

☐ None ☐ Elementary school diploma ☐ Middle school diploma

☐ High school diploma ☐ University degree (BSc/MSc/other)

Job: ____

Smoking status:

☐ Current smoker ☐ Nover smoked ☐ Former smoker

Alcohol consumption:

☐ No consumption

☐ Wine, beer, aperitifs:
The consumption is:
□ Occasional consumption
□ Habitual consumption → n° of glasses per day: ____

☐ Liquor and/or spirits:
The consumption is:
□ Occasional consumption
□ Habitual consumption → n° of glasses per day: ____

Do you exercise regularly?

☐ Yes ☐ No

How do you usually spend your free time? (*watching TV, reading, using smartphone, playing videogames, walking, gardening, dog walking, cycling, etc…*)

____

____

____

____

HOUSING SCHEDULE OF THE FAMILY

Please, answer the following questions by ticking only one box (by the parent)

House type:<br>□ Indipendent □ Flat/Apartment<br>⇨ If flat/apartment, on which floor is located? ______________________________
Do you live in a privately owned house?<br>□ Yes □ No
Is there an elevator in the building?<br>□ Yes □ No
Do you generally encourage your child(ren) to take the stairs?<br>□ Yes □ No
Does the child(ren) take the stairs regularly?<br>□ Yes □ No
Does the house/building have a garden/play area?<br>□ Yes □ No
Are there gardens/play areas nearby?<br>□ Yes □ No
Are there sports/recreational facilities near the house?<br>□ Yes □ No
Are cycle paths present in the neighborhood?<br>□ Yes □ No
Does the child ride a bike, regularly or just on weekends?<br>□ Yes □ No
The distance from the house to school is reachable:<br>Please, note: report only 1 response<br>□ It can also be reached on foot/bike □ Can only be reached by car or public transportation
How does the child(ren) usually get to school?<br>Please, note: report only 1 response<br>□ By foot □ By bicycle<br>□ By school bus/public transport<br>□ By car □ Other: ____________________
Are there fast-food restaurants/pizzerias/takeaways near the house?<br>□ Yes □ No
Are there markets/supermarkets with fresh food products near the house?<br>□ Yes □ No

MEAL ORGANIZATION SHEET

To be completed by the parents, concerning the family habits

How often do you consume the main meals (lunch/dinner) out of home per week?			
□ < 1	□ 1	□ 2 o 3	□ ≥ 4
How often do you eat at fast-food restaurants/pizzerias/takeaways or food from fast-food restaurants/pizzerias/takeaways per week?			
□ <1	□ 1	□ 2 o 3	□ ≥ 4
How often do you consume ready-to-eat or pre-packaged foods per week?			
□ < 1	□ 1	□ 2 o 3	□ ≥ 4
Do people in your family eat meals while watching TV?			
□ Yes		□ Occasionally	
□ Often		□ No	
Are snacks consumed in your family while watching TV, playing computer or video games?			
□ Yes		□ Occasionally	
□ Often		□ No	
Are there snacks, pretzels, chips, sweets, etc. present in the house pantry?			
□ Yes		□ No	
If you marked yes:			
Does the child have free access to these foods?		□ Yes	□ No
Are cookies or sweets used in your family as a reward for the child(ren)?			
□ Yes		□ No	

SHEET ON THE CHILD'S HABITS

To be completed by the parents

PHYSICAL ACTIVITY

Does your child engage in unplanned physical activity in his or her free time (going to the park, biking/skateboarding/skating, etc.)?

□ Yes □ No

If yes, please indicate which ones: ______________________________________

On average, how many minutes per day during the week? ___________________________

Does your child participate in organized/instructor-led physical activity?

□ Yes □ No

If yes, indicate which, how often per week and for how many hours:

	Times per week				Hours per week
☐ Volleyball	□1	□ 2	□ 3	□ ≥4	
☐ Basketball	□1	□ 2	□ 3	□ ≥4	
☐ Soccer	□1	□ 2	□ 3	□ ≥4	
☐ Rugby	□1	□ 2	□ 3	□ ≥4	
☐ Dance	□1	□ 2	□ 3	□ ≥4	
☐ Gymnastics	□1	□ 2	□ 3	□ ≥4	
☐ Rythmic gymnastics	□1	□ 2	□ 3	□ ≥4	
☐ Karate/martial arts	□1	□ 2	□ 3	□ ≥4	
☐ Swimming	□1	□ 2	□ 3	□ ≥4	
☐ Synchronized swimming	□1	□ 2	□ 3	□ ≥4	
☐ Water polo	□1	□ 2	□ 3	□ ≥4	
☐ Athletics	□1	□ 2	□ 3	□ ≥4	
☐ Circus academy	□1	□ 2	□ 3	□ ≥4	
☐ Others: _______________	□1	□ 2	□ 3	□ ≥4	

SCREEN TIME	
**Does the child have a TV for personal use in his/her room?**	
□ Yes	□ No
**Does the child have a computer/tablet/smartphone for personal use?**	
□ Yes	□ No
**Does the child have a game console for personal use?**	
□ Yes	□ No
**In a school day, how many hours does the child spend watching television and/or playing video games and/or using the computer (for non-scholastic purposes) on average?**	
□ < 1 hour	□ About 1 hour
□ About 2 hours	□ About 3 hours
□ About 4 hours	□ More than 5 hours

SLEEPING TIME	
**Does the child have a bedtime routine?**	
□ Yes	□ No
**Does the child have a scheduled bedtime?**	
□ Yes	□ No
**How many hours does the child sleep per night?**	
□ Less than 9 hours	□ From 9 to 12 hours
□ More than 12 hours	

CHILD'S EATING HABITS

To be completed by the parents

What meals does your child usually eat?

☐ Breakfast ☐ Mid-morning snack

☐ Lunch ☐ Mid-afternoon snack

☐ Dinner ☐ Snack before bed time

Does the child use the school canteen?

☐ Yes, every day ☐ Yes, only some days

☐ No

Does the child consume whole grain foods such as pasta, rice and/or other cereals?

☐ Yes ☐ No

If yes, please indicate how many times per week: ________________________________

The child uses to sweeten drinks (tea, milk, etc...)

☐ Sugar; how many spoons? _____________

☐ Nesquik, how many spoons? _____________

☐ Honey, how many spoons? _____________

☐ Other: __________________ , how many spoons? _____________

☐ Nothing

HIDRATION

How many liters of water does the child drink during the day?

☐ Less than 1 litre ☐ Between 1 and 1.5 litres

☐ Between 1.5 and 2 litres ☐ More than 2 litres

EVALUATION OF ADHERENCE TO THE MEDITERRANEAN DIET (KIDMED QUESTIONNAIRE)

☒ Please, answer the following questions by marking one box only

**1**	Does the child consume a fruit or fresh juice every day?	☐ Yes	☐ No
**2**	Does the child eat a second portion of fruit every day?	☐ Yes	☐ No
**3**	Does the child consume vegetables, cooked or raw, regularly, once per day?	☐ Yes	☐ No
**4**	Does the child consume vegetables, cooked or raw, more than once per day?	☐ Yes	☐ No
**5**	Does the child consume fish regularly (at least 2-3 times per week)?	☐ Yes	☐ No
**6**	Does the child go to fast food restaurants/pizzeria/takeaways more than once per week?	☐ Yes	☐ No
**7**	Does the child like legumes and eat them more than once per week?	☐ Yes	☐ No
**8**	Does the child consume pasta and rice every day (5 or more times per week)?	☐ Yes	☐ No
**9**	Does the child have cereal grains or bread for breakfast?	☐ Yes	☐ No
**10**	Does the child regularly consume nuts (at least 2-3 times per week)?	☐ Yes	☐ No
**11**	Is olive oil used at home for the preparation of dishes consumed by the child?	☐ Yes	☐ No
**12**	Does the child skip breakfast?	☐ Yes	☐ No
**13**	Does the child consume dairy products for breakfast (yogurt, milk, etc.)?	☐ Yes	☐ No
**14**	Does the child consume baked or pastry products for breakfast?	☐ Yes	☐ No
**15**	Does the child consume two yoghurt and/or cheese (40 g) per day?	☐ Yes	☐ No
**16**	Does the child consume candies or sweets several times per day?	☐ Yes	☐ No

ULTRA-PROCESSED FOODS CONSUMPTION

<u>In the last week, how many times did the child consume the foods listed below?</u>

☒ Please, answer the following questions by marking one box only

Packaged and industrialized bread (e.g. Pan carrè, Pan Bauletto...)

□ Never/Occasionally □ Less than once a week □ 2 times per week
□ 3 times per week □ 4 times per week □ 5 times per week
□ 6 times per week □ 7 times per week

Sausages and other meat products

□ Never/Occasionally □ Less than once a week □ 2 times per week
□ 3 times per week □ 4 times per week □ 5 times per week
□ 6 times per week □ 7 times per week

Packaged ready-to-eat meals

□ Never/Occasionally □ Less than once a week □ 2 times per week
□ 3 times per week □ 4 times per week □ 5 times per week
□ 6 times per week □ 7 times per week

Breakfast cereals

□ Never/Occasionally □ Less than once a week □ 2 times per week
□ 3 times per week □ 4 times per week □ 5 times per week
□ 6 times per week □ 7 times per week

Candies

□ Never/Occasionally □ Less than once a week □ 2 times per week
□ 3 times per week □ 4 times per week □ 5 times per week
□ 6 times per week □ 7 times per week

Cookies

□ Never/Occasionally □ Less than once a week □ 2 times per week
□ 3 times per week □ 4 times per week □ 5 times per week
□ 6 times per week □ 7 times per week

Desserts, focaccias and cakes

□ Never/Occasionally □ Less than once a week □ 2 times per week
□ 3 times per week □ 4 times per week □ 5 times per week
□ 6 times per week □ 7 times per week

Potato chips

□ Never/Occasionally □ Less than once a week □ 2 times per week
□ 3 times per week □ 4 times per week □ 5 times per week
□ 6 times per week □ 7 times per week

In the last week, how many times did the child consume the foods listed below?

☒ Please, answer the following questions by marking one box only

Fruit juices

- ◻ Never/Occasionally
- ◻ Less than once a week
- ◻ 2 times per week
- ◻ 3 times per week
- ◻ 4 times per week
- ◻ 5 times per week
- ◻ 6 times per week
- ◻ 7 times per week

Milk-based drinks

- ◻ Never/Occasionally
- ◻ Less than once a week
- ◻ 2 times per week
- ◻ 3 times per week
- ◻ 4 times per week
- ◻ 5 times per week
- ◻ 6 times per week
- ◻ 7 times per week

Salty snacks (e.g. pretzels, peanuts ...)

- ◻ Never/Occasionally
- ◻ Less than once a week
- ◻ 2 times per week
- ◻ 3 times per week
- ◻ 4 times per week
- ◻ 5 times per week
- ◻ 6 times per week
- ◻ 7 times per week

Frozen pizza

- ◻ Never/Occasionally
- ◻ Less than once a week
- ◻ 2 times per week
- ◻ 3 times per week
- ◻ 4 times per week
- ◻ 5 times per week
- ◻ 6 times per week
- ◻ 7 times per week

Margarine and other spreads

- ◻ Never/Occasionally
- ◻ Less than once a week
- ◻ 2 times per week
- ◻ 3 times per week
- ◻ 4 times per week
- ◻ 5 times per week
- ◻ 6 times per week
- ◻ 7 times per week

Ready-made sauces, condiments and sauces

- ◻ Never/Occasionally
- ◻ Less than once a week
- ◻ 2 times per week
- ◻ 3 times per week
- ◻ 4 times per week
- ◻ 5 times per week
- ◻ 6 times per week
- ◻ 7 times per week

Ready-made dessert

- ◻ Never/Occasionally
- ◻ Less than once a week
- ◻ 2 times per week
- ◻ 3 times per week
- ◻ 4 times per week
- ◻ 5 times per week
- ◻ 6 times per week
- ◻ 7 times per week

Soy and other milk substitute drinks

- ◻ Never/Occasionally
- ◻ Less than once a week
- ◻ 2 times per week
- ◻ 3 times per week
- ◻ 4 times per week
- ◻ 5 times per week
- ◻ 6 times per week
- ◻ 7 times per week
