# Supplementary material for: Multidimensional School-Based and Family-Involved Interventions to Promote a Healthy and Sustainable Lifestyle (LIVELY) for Childhood Obesity Prevention: Study Protocol
Source: JMIR Res Protoc. 2024 Oct 30;13:e57509. doi: 10.2196/57509 (PMC11561434; doi:10.2196/57509)

Mu**L**tidimensional school-based and family **I**nvolved inter**V**entions, to promote a h**E**althy and sustainable **L**ifest**Y**le for the childhood obesity primary prevention

2. MICRONUTRIENTS

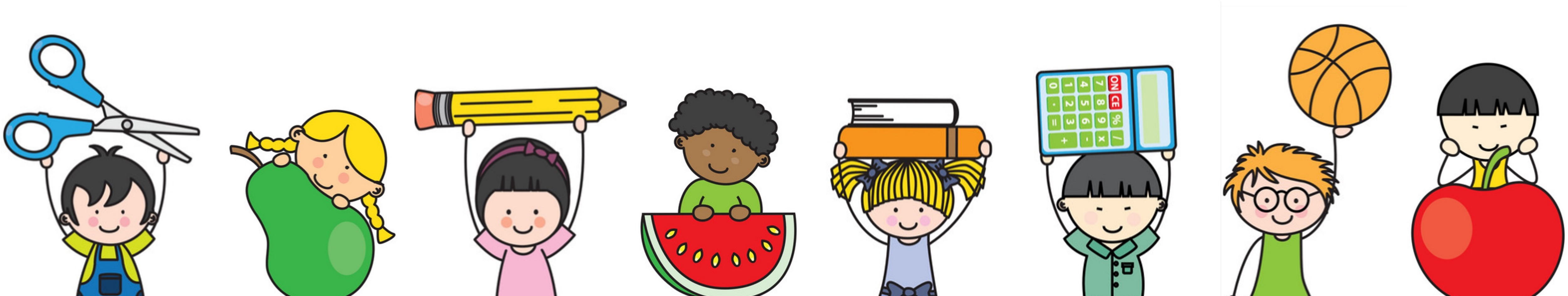

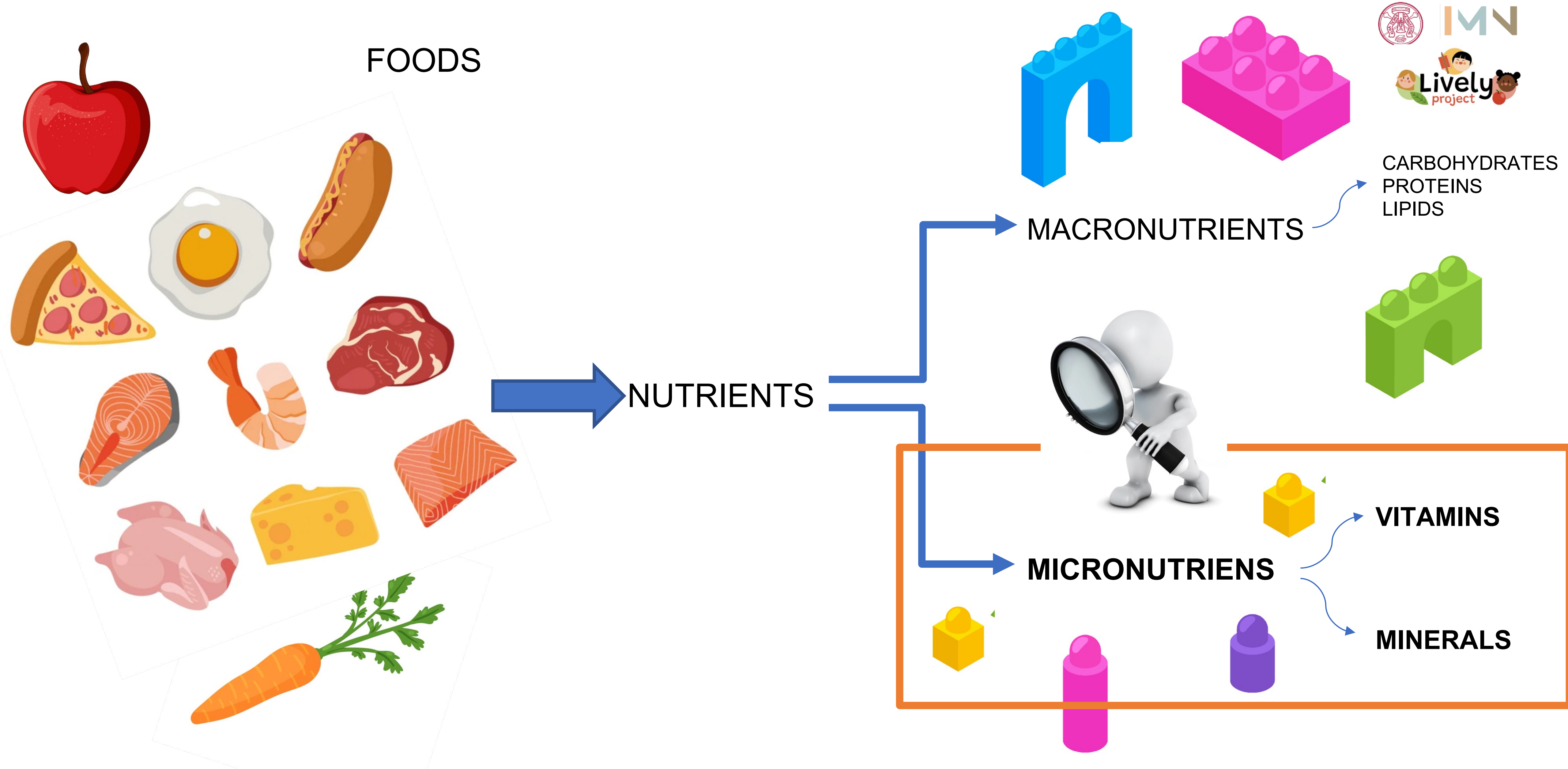
FOODS
NUTRIENTS
MACRONUTRIENTS
CARBOHYDRATES
PROTEINS
LIPIDS
MICRONUTRIENS
VITAMINS
MINERALS
Lively project

Finanziato dall'Unione europea NextGenerationEU
Ministero dell'Università e della Ricerca
Italiadomani PIANO NAZIONALE DI RIPRESA E RESILIENZA
onfoods

THE 8 SUPERHEROS: VITAMINS

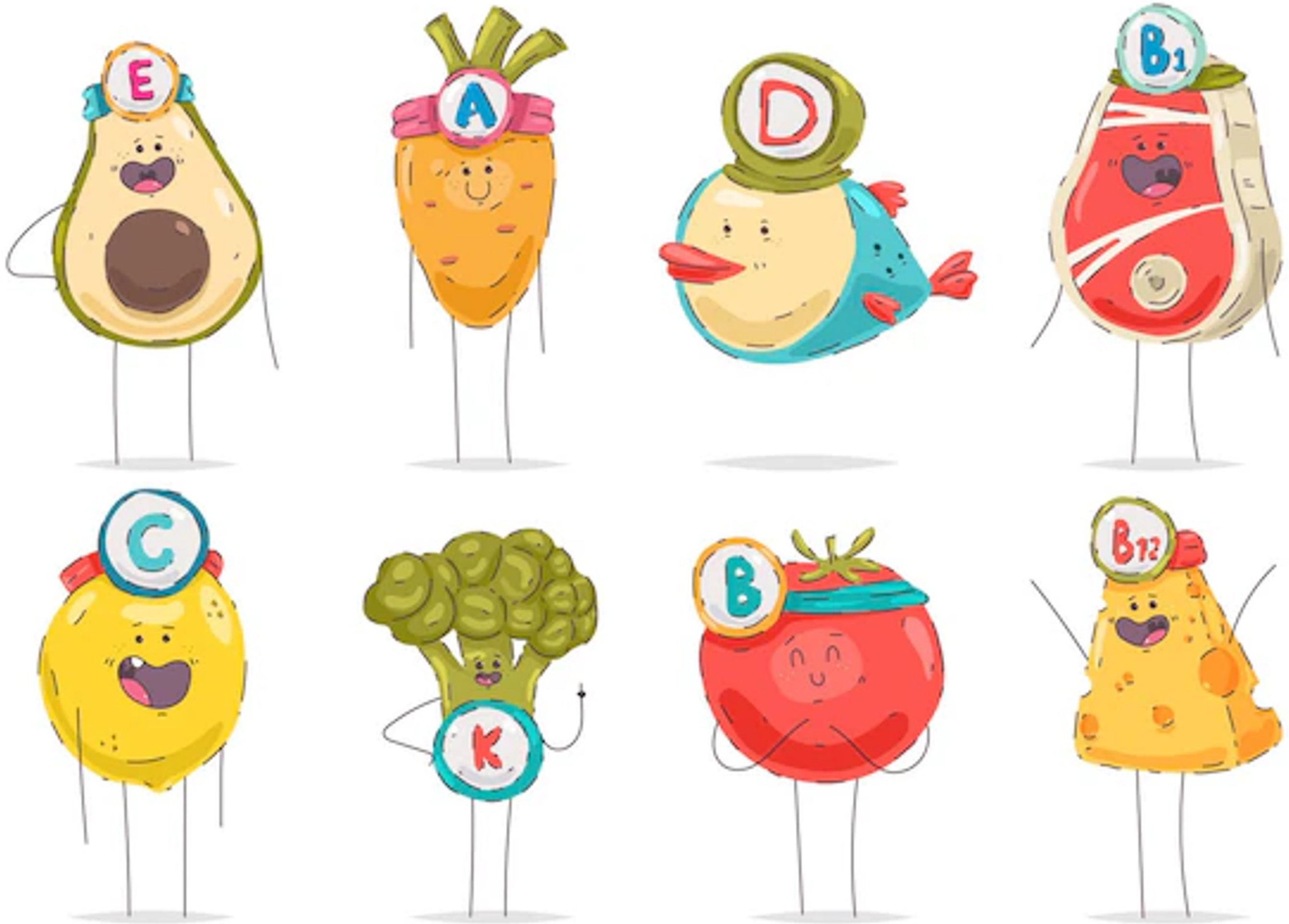

VITAMINS WHICH «MELT» IN FAT (LIPOSOLUBLE)

- VITAMIN A
- VITAMIN D
- VITAMIN K
- VITAMIN E

VITAMINS WHICH «MELT» IN WATER (HYDROSOLUBLE)

- GROUP B VITAMINS
- VITAMIN C

LIPOSOLUBLE = THEY DISSOLVE IN FAT

HYDROSOLUBLE = THEY DISSOLVE IN WATER

Finanziato dall'Unione europea
NextGenerationEU
Ministero dell'Università e della Ricerca
Italiadomani
PIANO NAZIONALE DI RIPRESA E RESILIENZA
onfoods

VITAMIN D

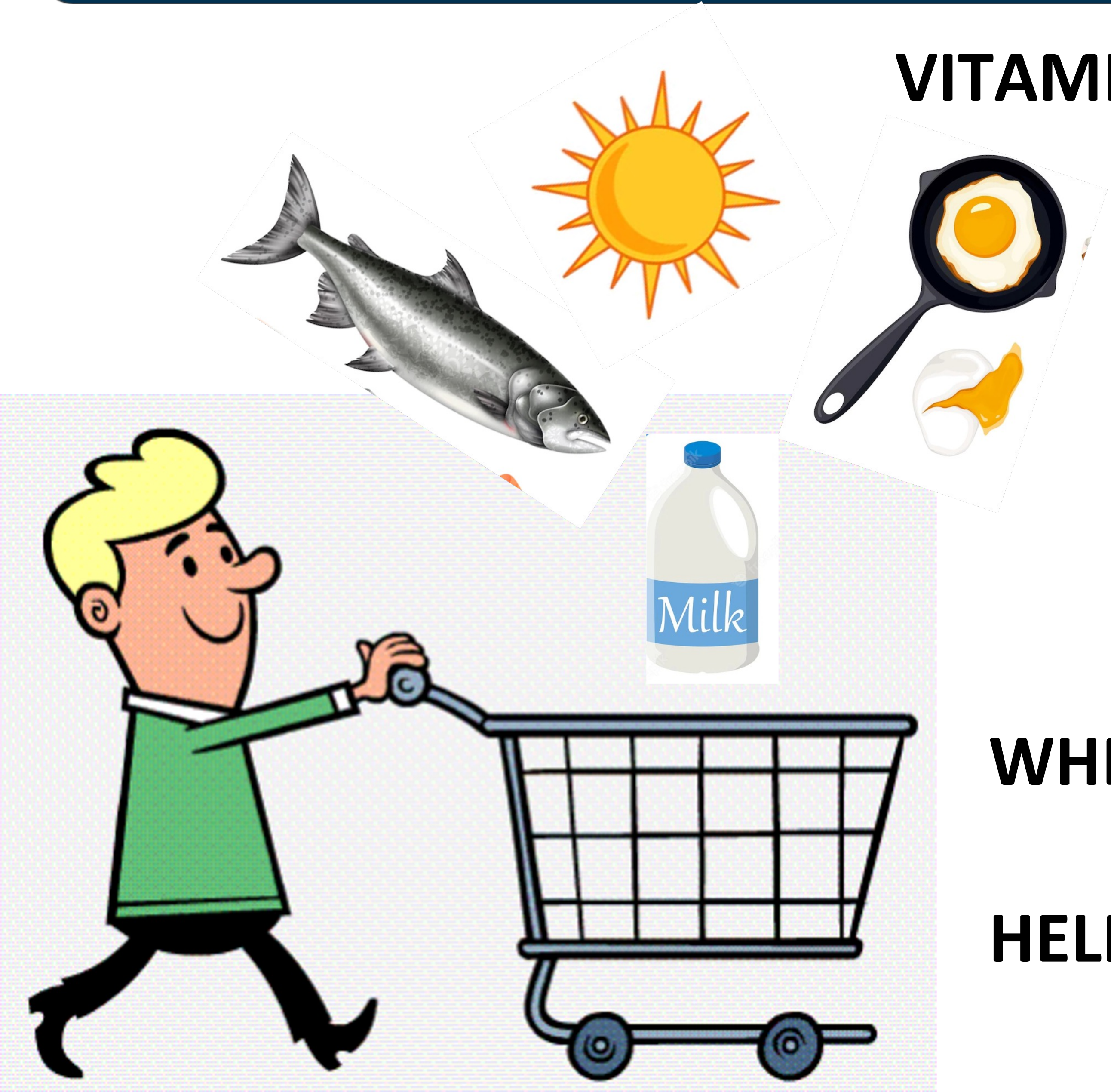

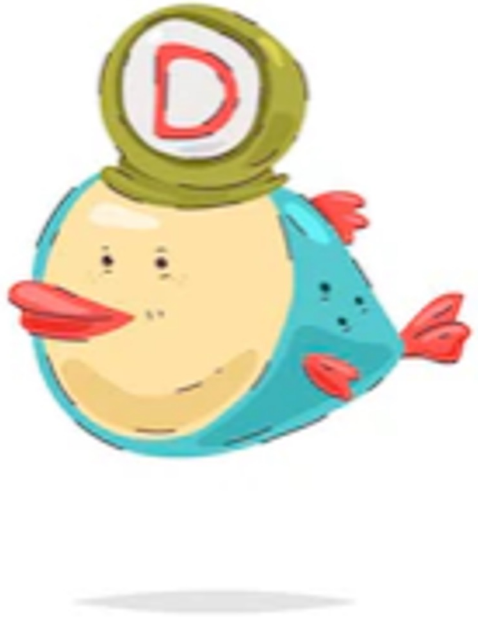

WHERE YOU CAN FIND VITAMIN D?

HELP FRED WITH THE GROCERIES!

Finanziato dall'Unione europea NextGenerationEU
Ministero dell'Università e della Ricerca
Italiadomani
onfoods

VITAMIN K

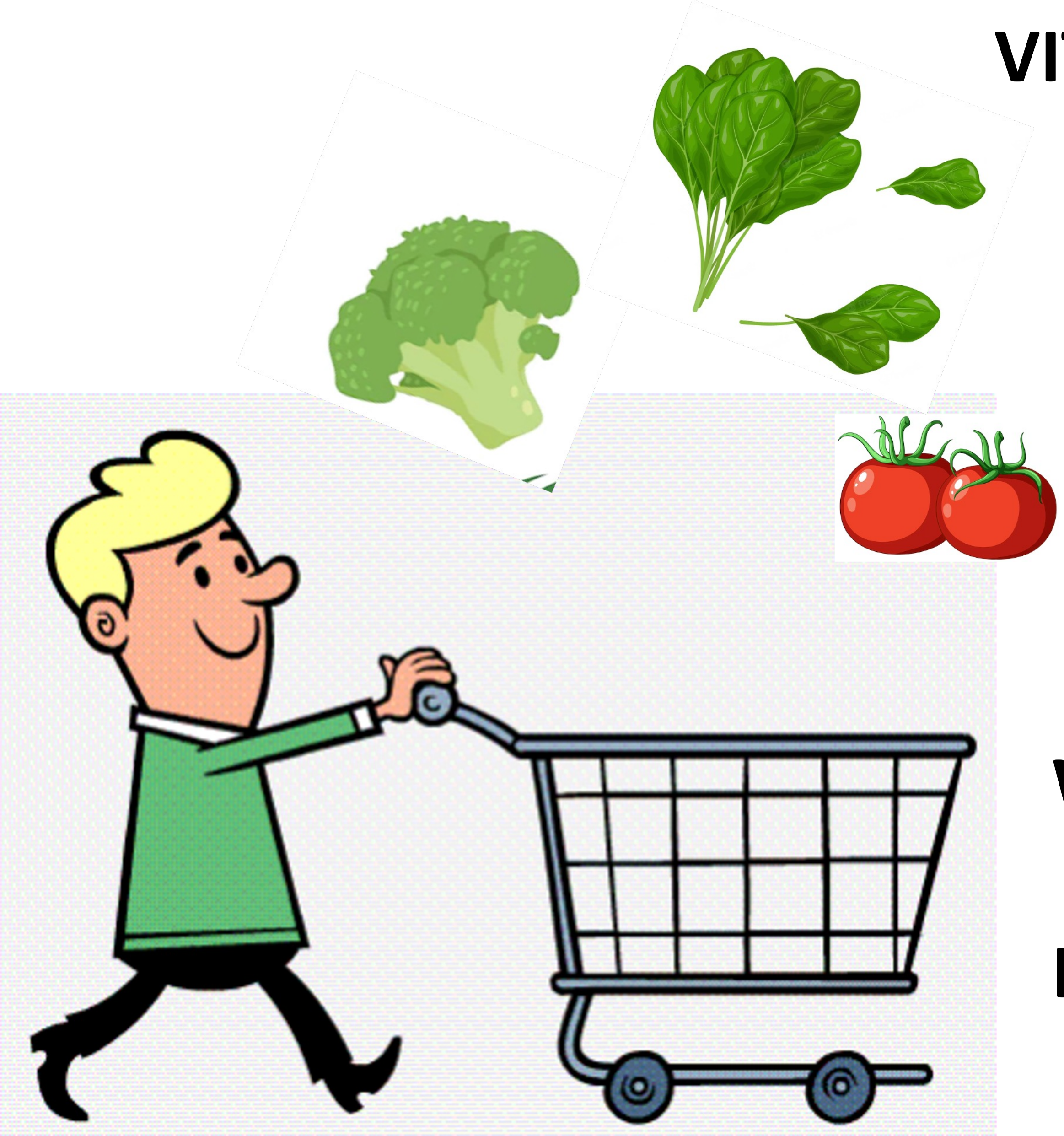

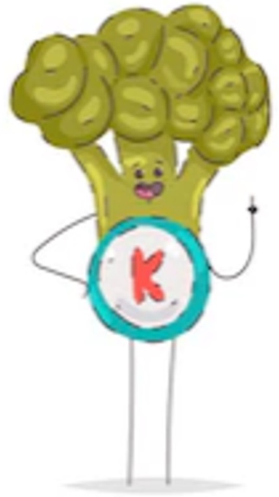

WHERE YOU CAN FIND VITAMIN K?

HELP FRED WITH THE GROCERIES!

Finanziato dall'Unione europea NextGenerationEU
Ministero dell'Università e della Ricerca
Italiadomani PIANO NAZIONALE DI RIPRESA E RESILIENZA
onfoods
Lively project

VITAMIN D

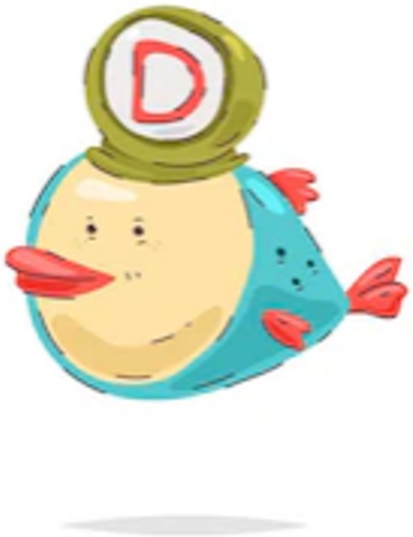

VITAMIN K

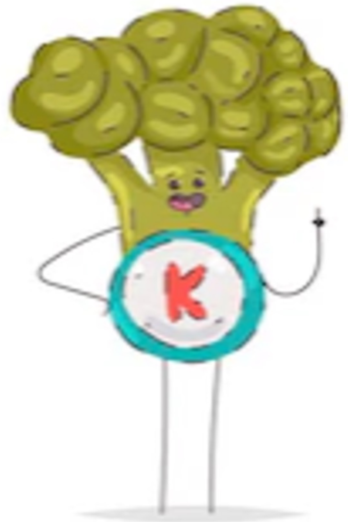

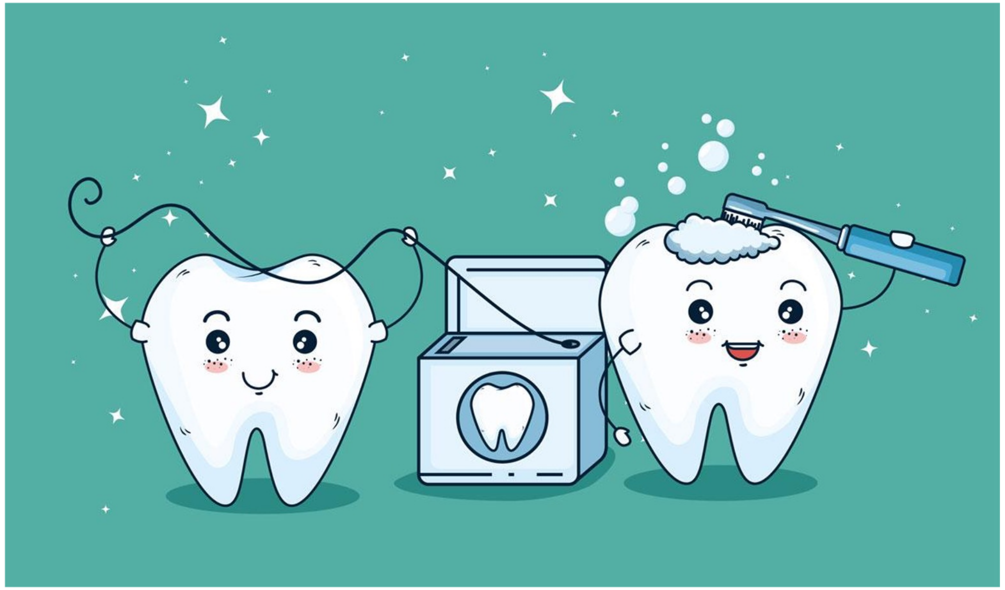

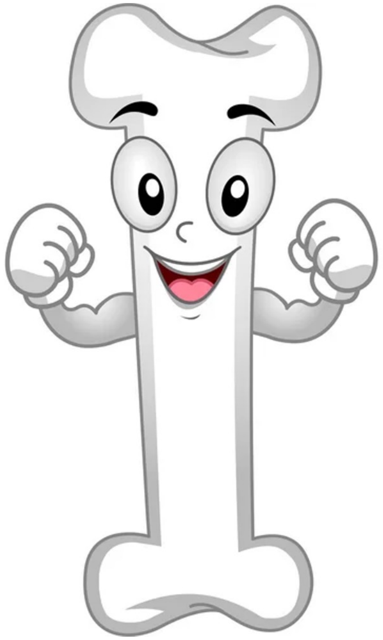

Finanziato dall'Unione europea NextGenerationEU
Ministero dell'Università e della Ricerca
Italiadomani
PIANO NAZIONALE DI RIPRESA E RESILIENZA
onfoods

VITAMIN A

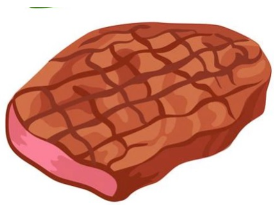
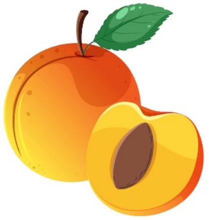
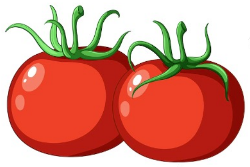
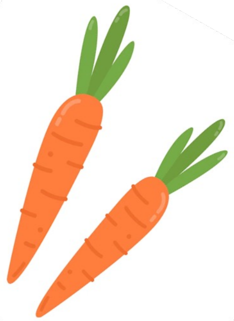
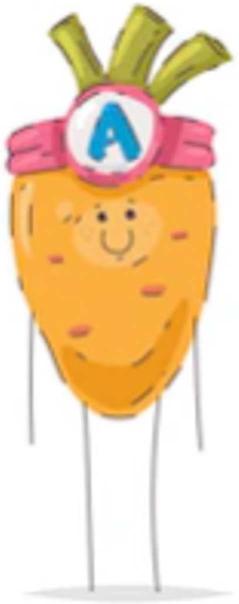

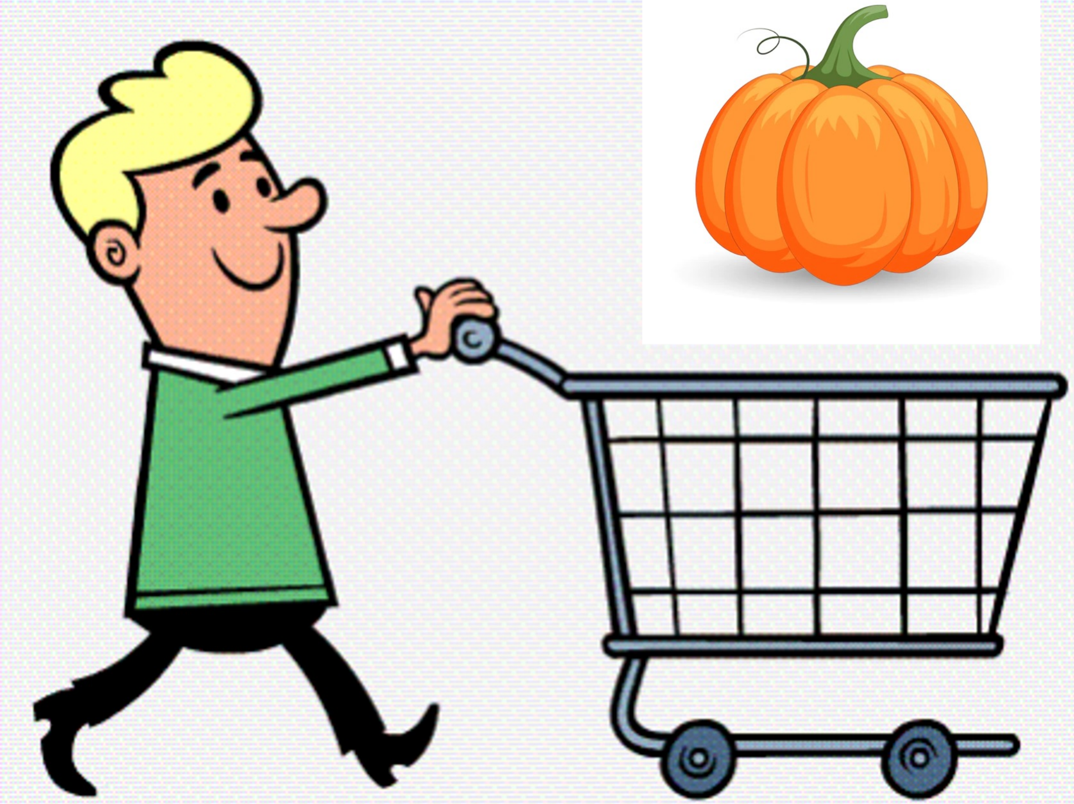

WHERE YOU CAN FIND VITAMIN A?

HELP FRED WITH THE GROCERIES!

VITAMIN A

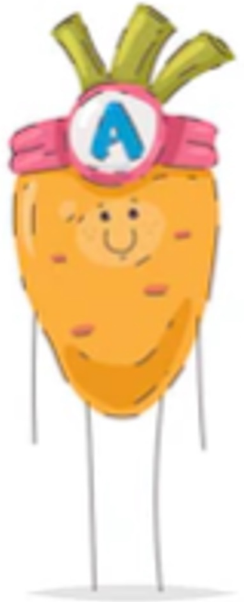

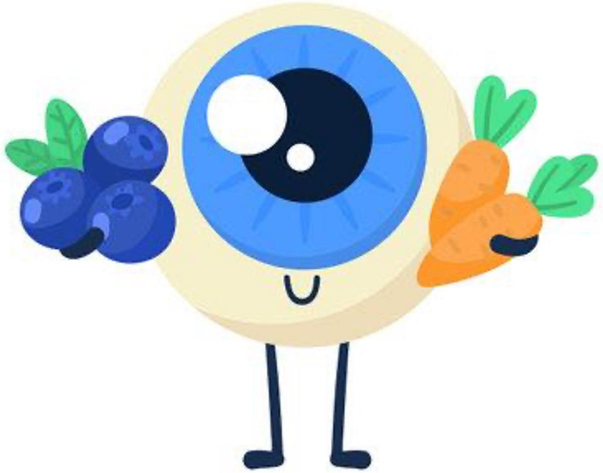

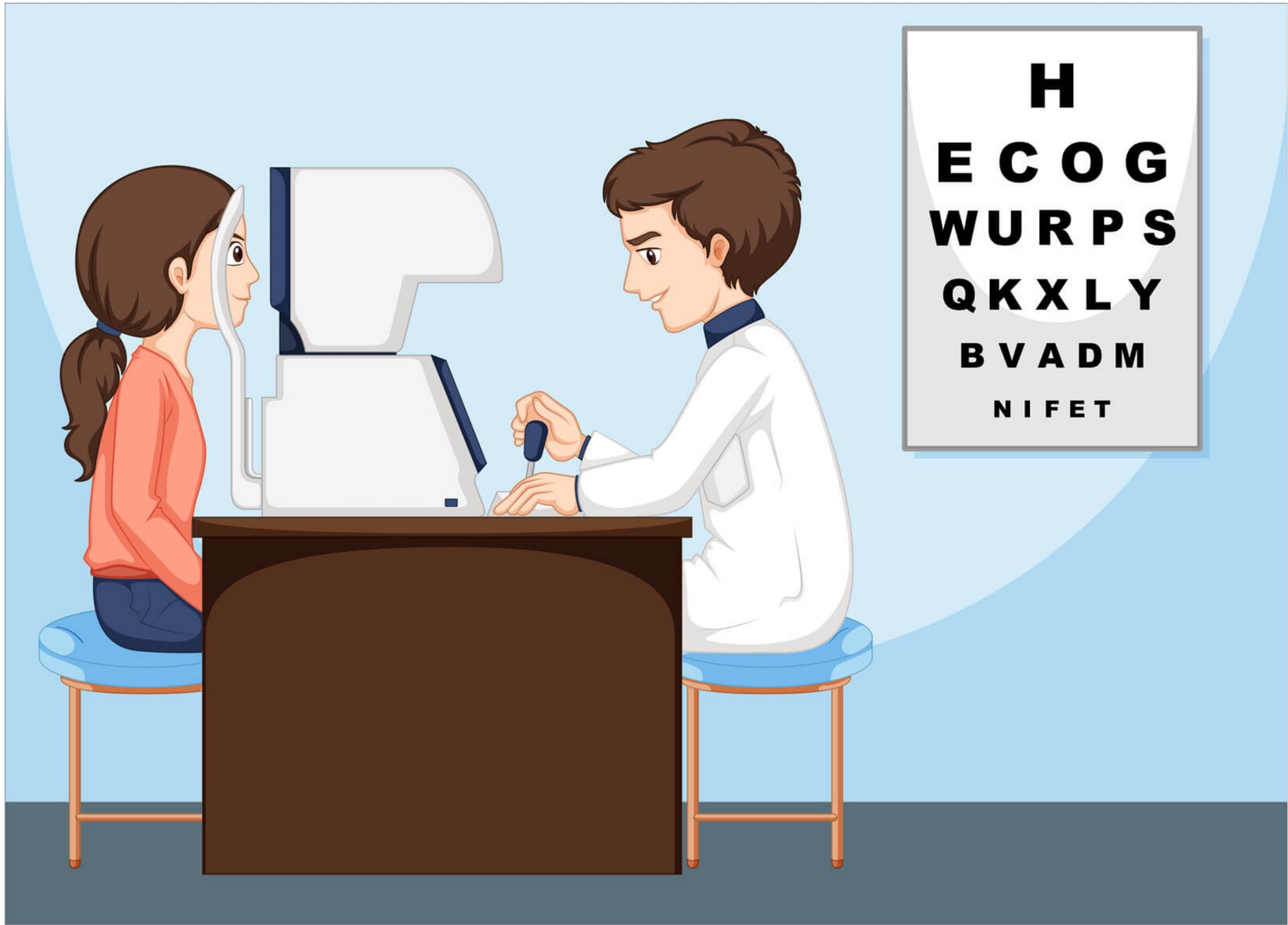

Finanziato dall'Unione europea
NextGenerationEU
Ministero dell'Università e della Ricerca
Italiadomani
onfoods

VITAMIN E

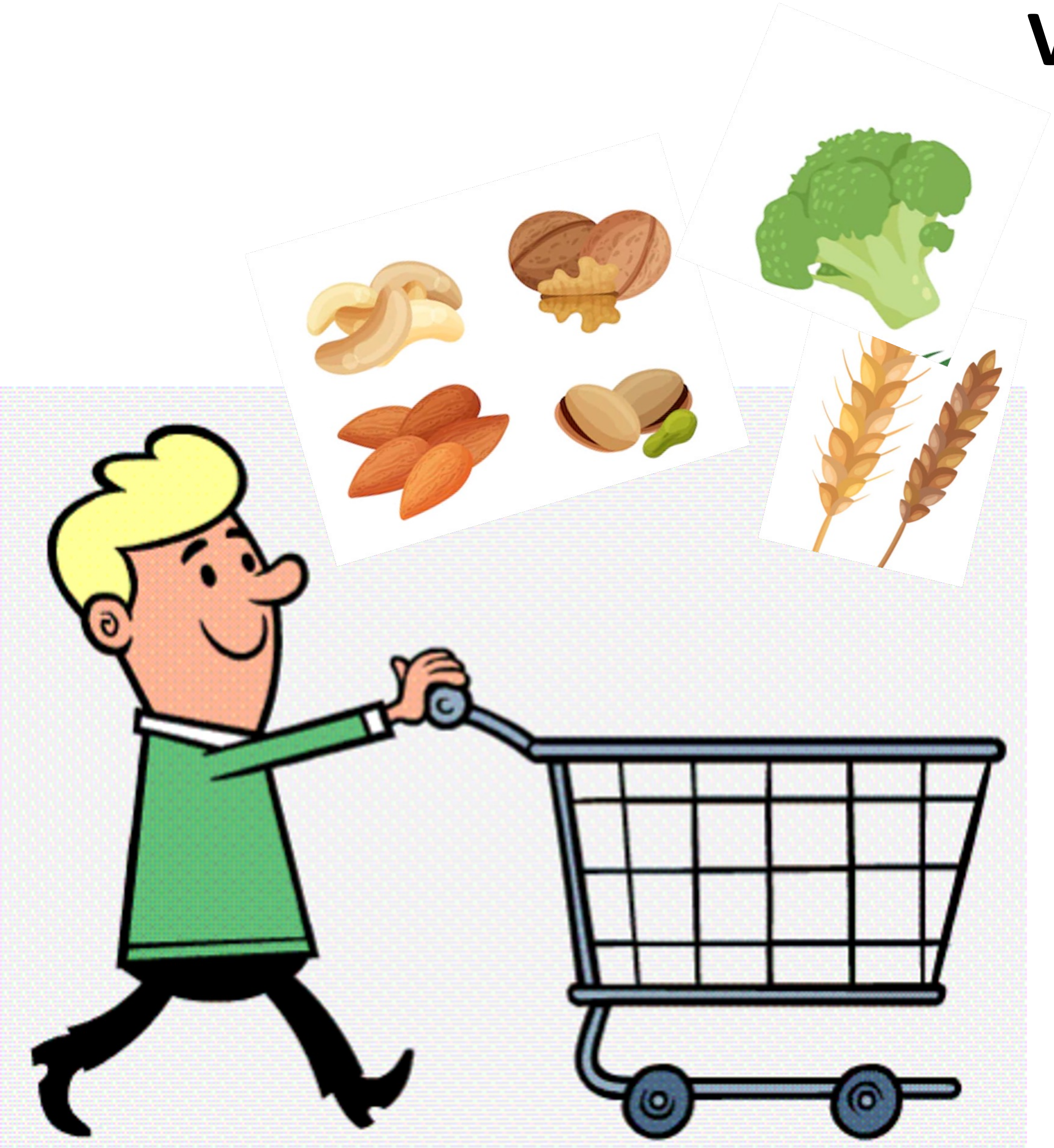

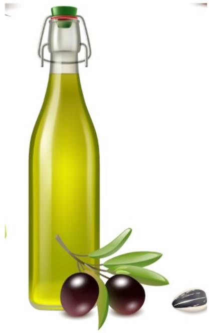

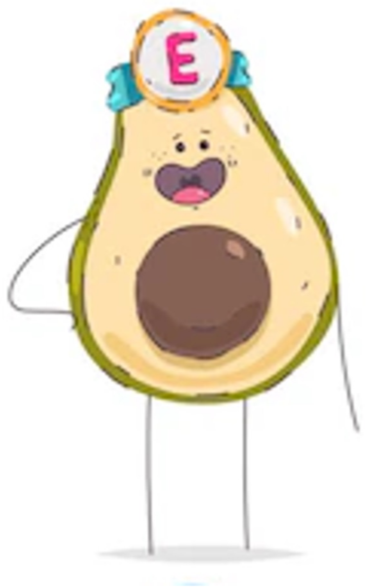

WHERE YOU CAN FIND VITAMIN E?

HELP FRED WITH THE GROCERIES!

VITAMIN C

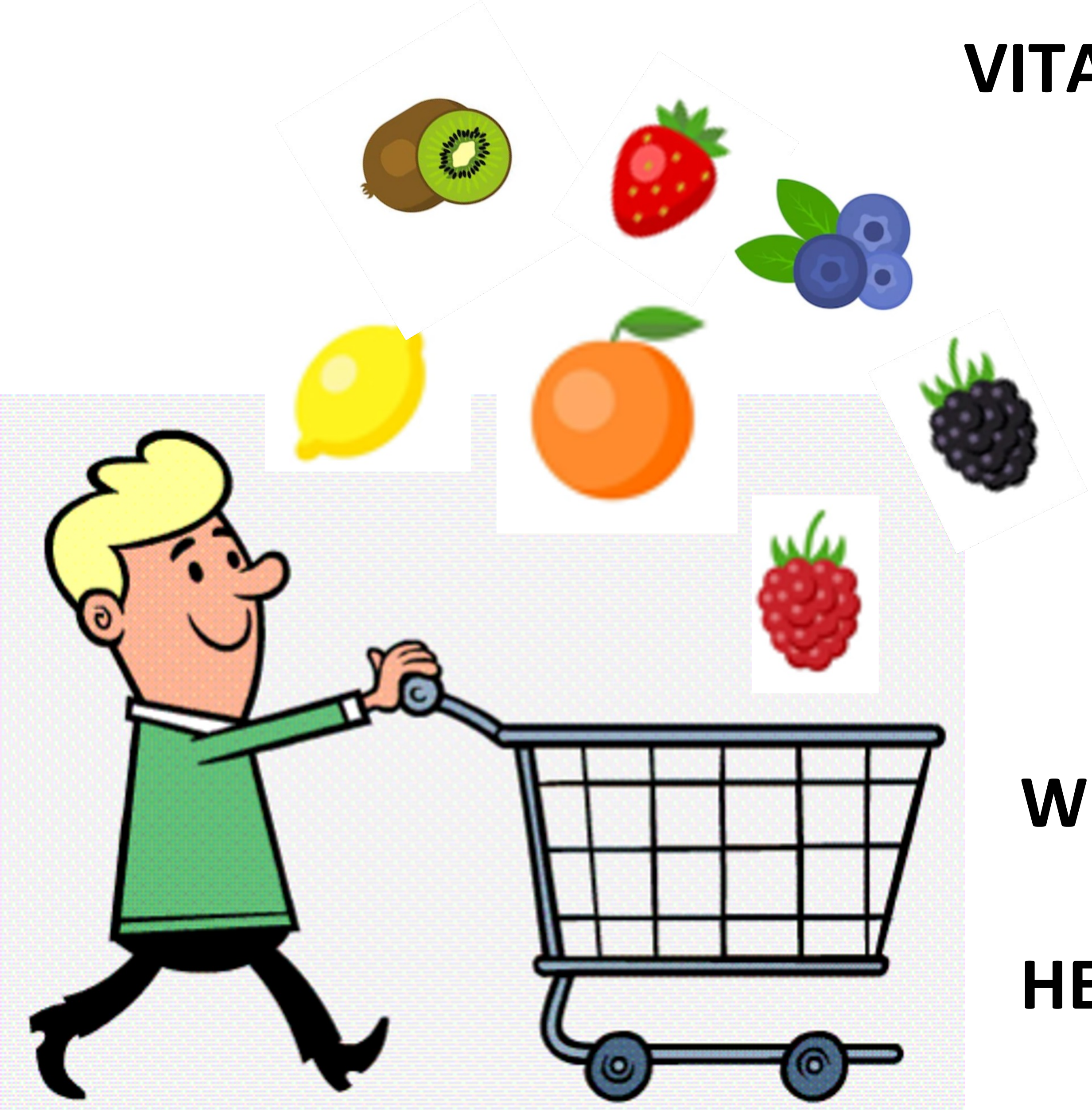

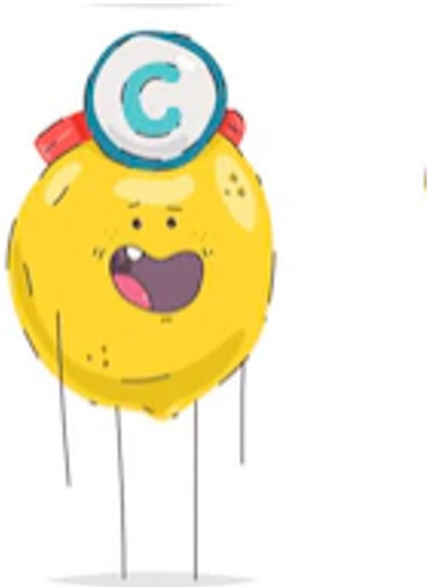

WHERE YOU CAN FIND VITAMIN C?

HELP FRED WITH THE GROCERIES!

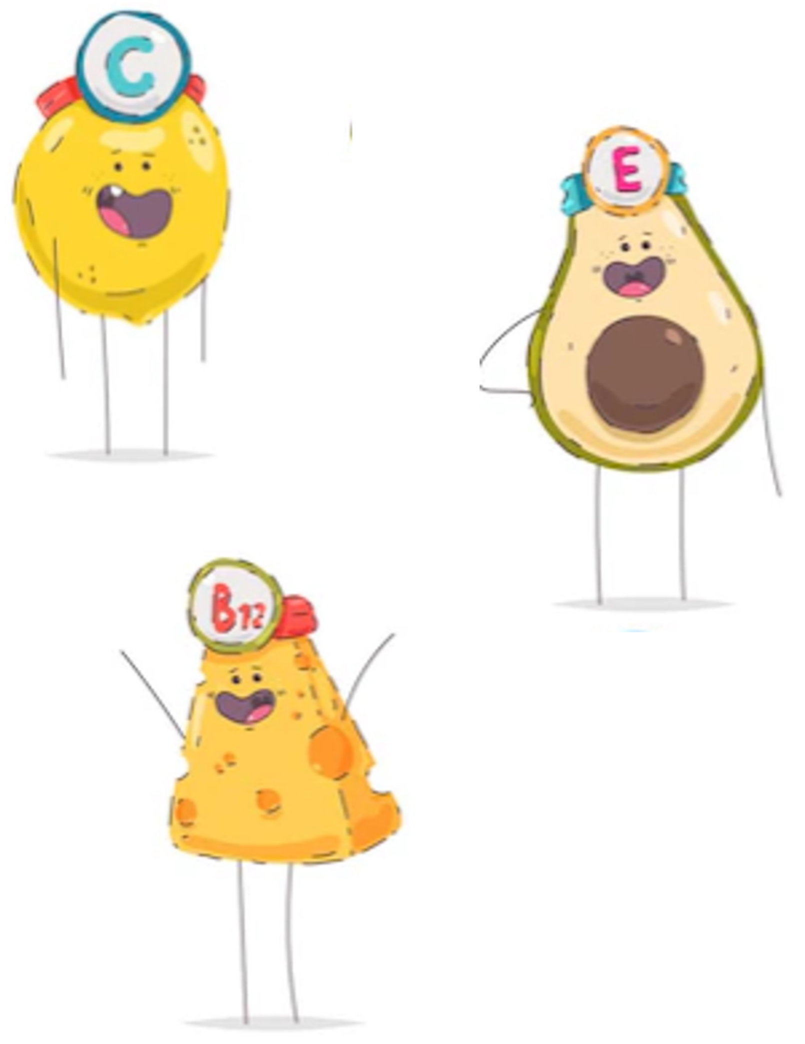

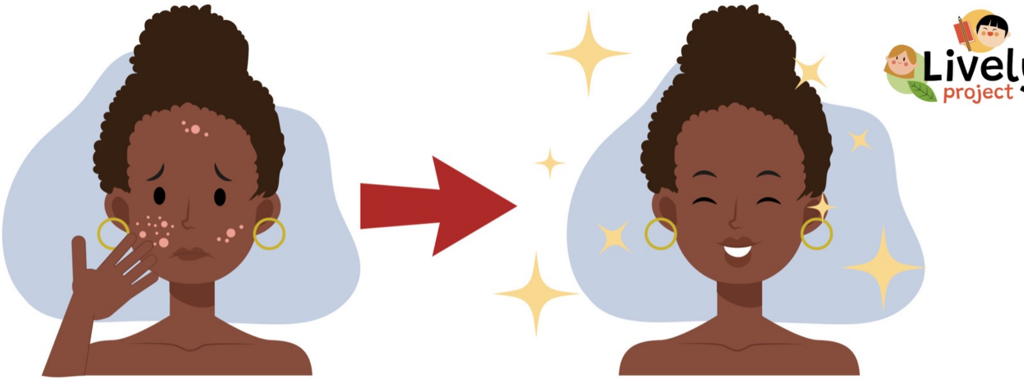

Lively project

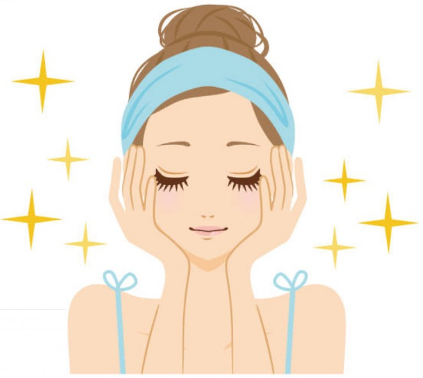

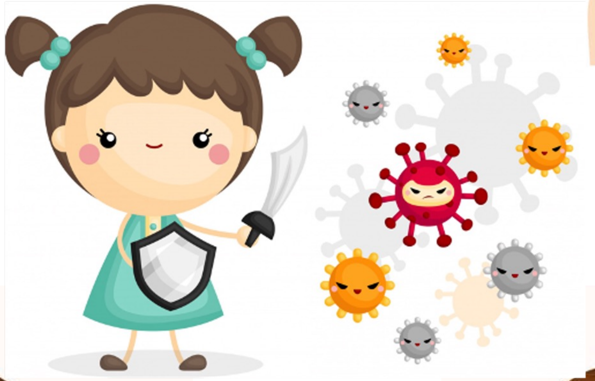

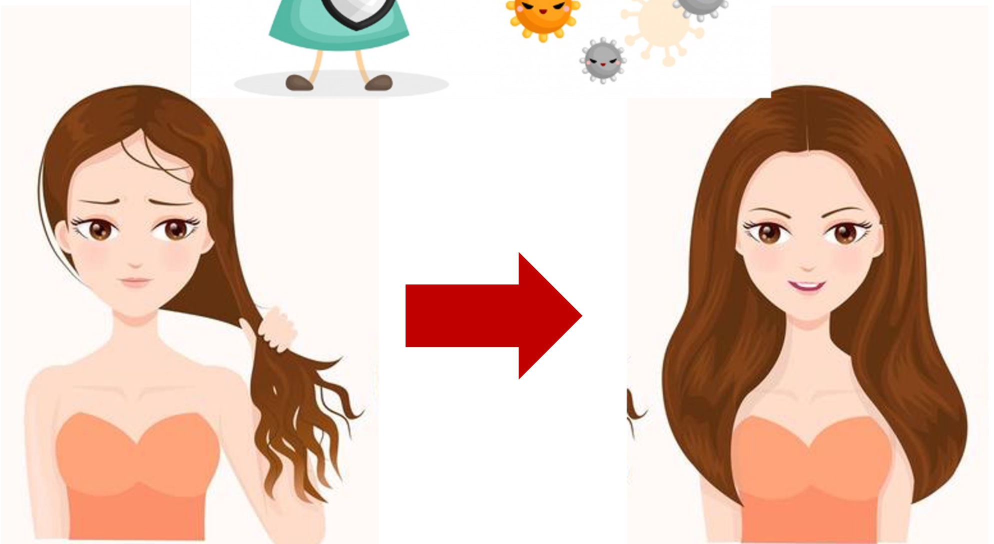

THE SEVEN MINERALS

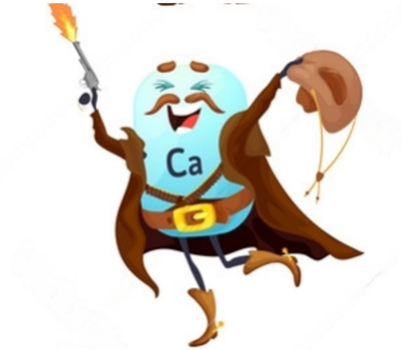

CALCIUM

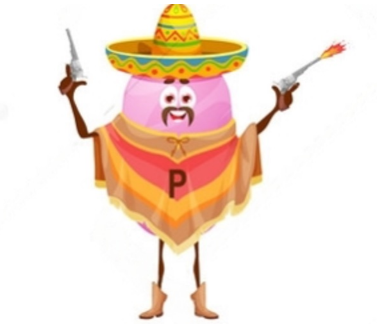

PHOSPHORUS

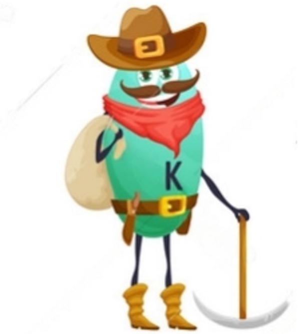

POTASSIUM

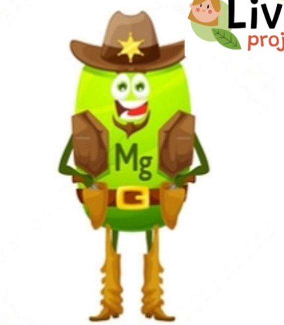

MAGNESIUM

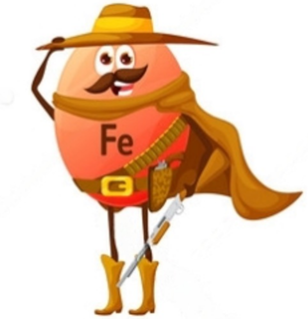

IRON

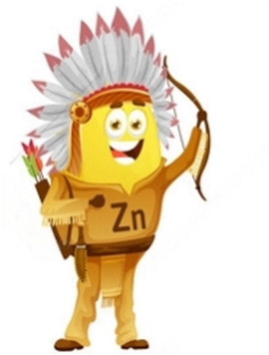

ZINC

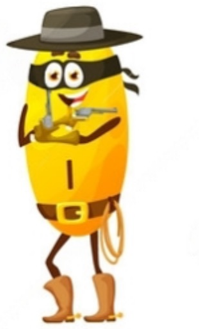

IODIUM

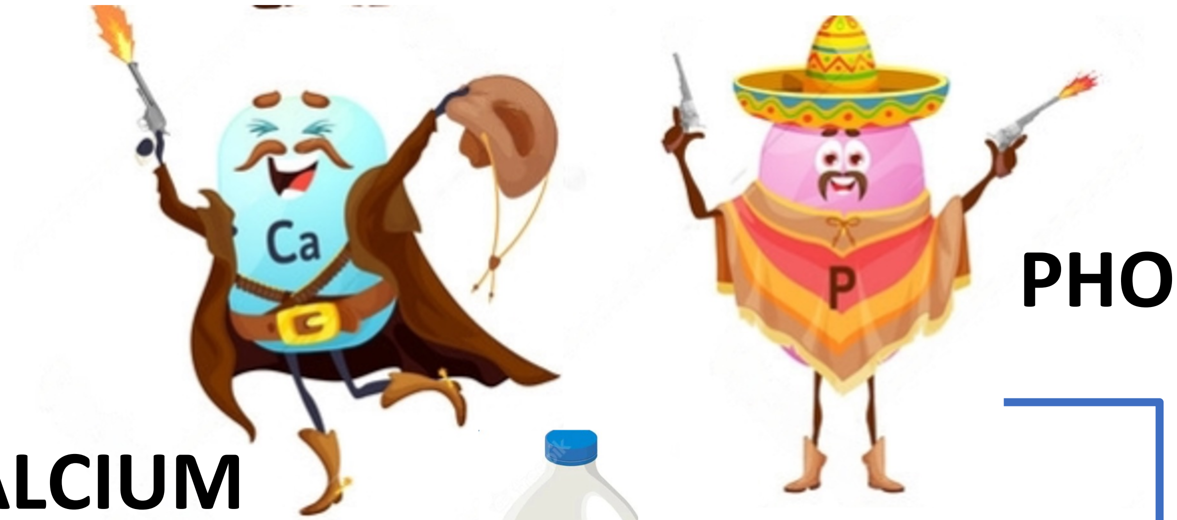

PHOSPHORUS

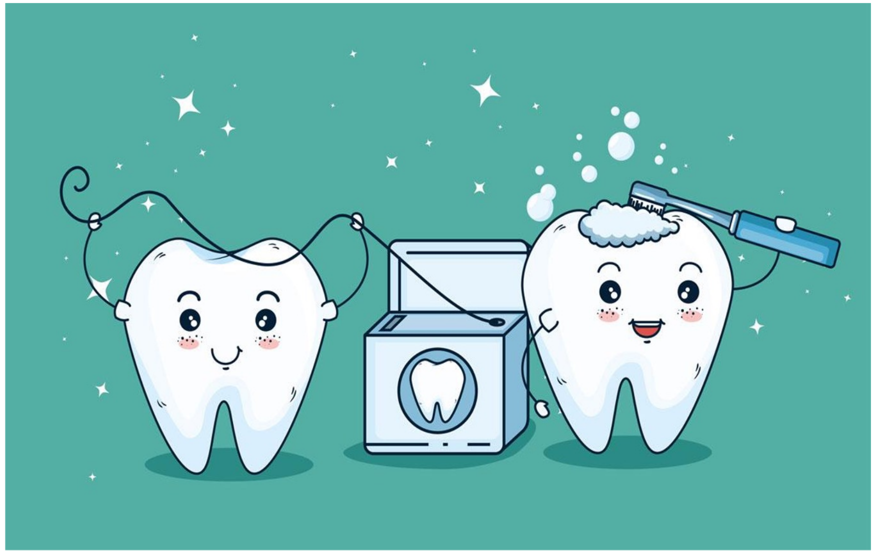

CALCIUM

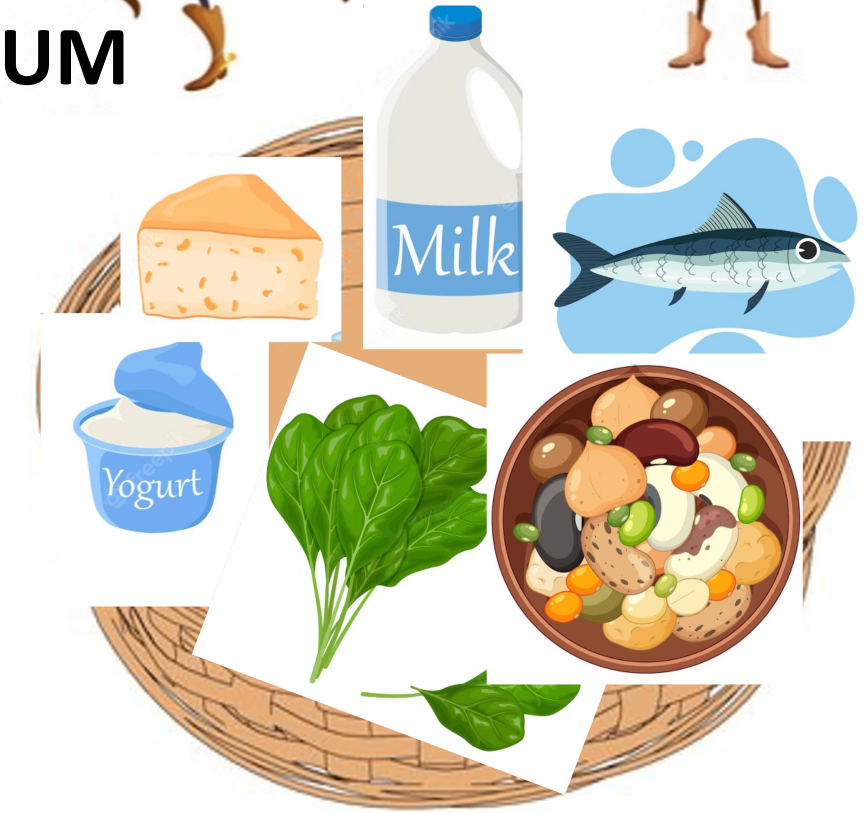

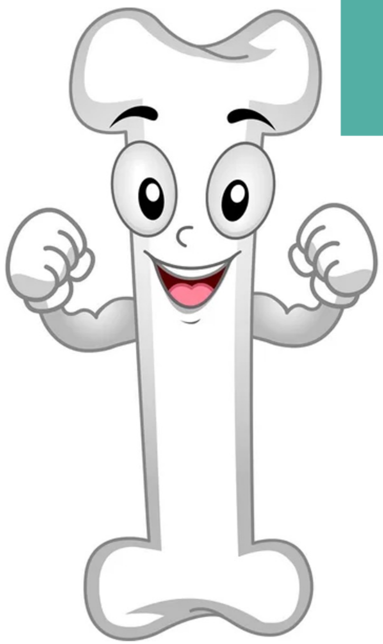

Finanziato dall'Unione europea NextGenerationEU
Ministero dell'Università e della Ricerca
Italiadomani PIANO NAZIONALE DI RIPRESA E RESILIENZA
onfoods

IRON

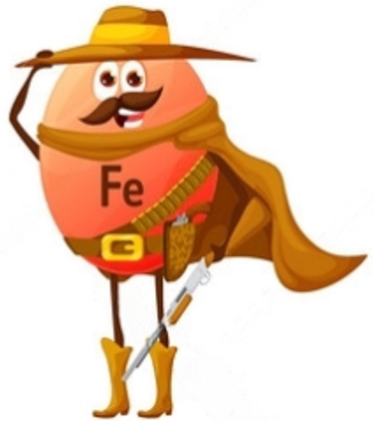

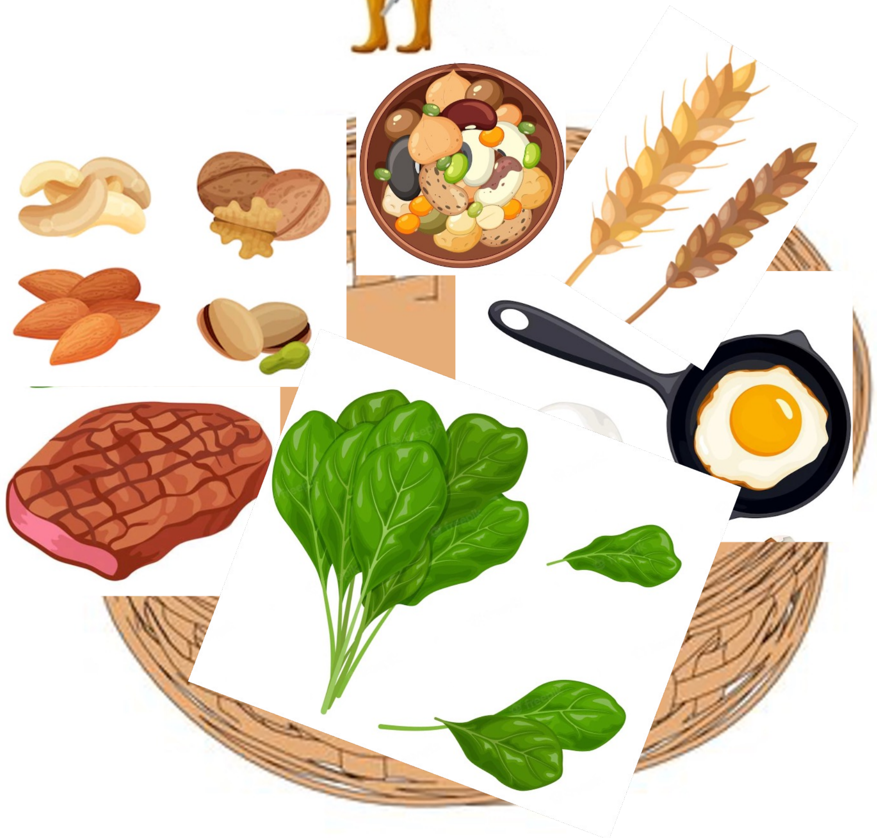

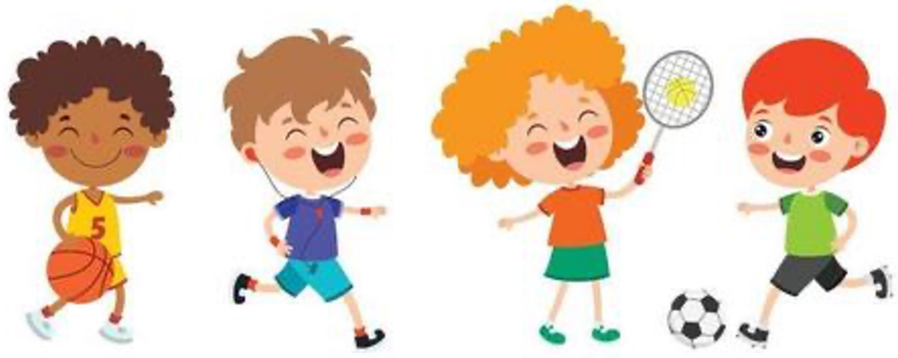

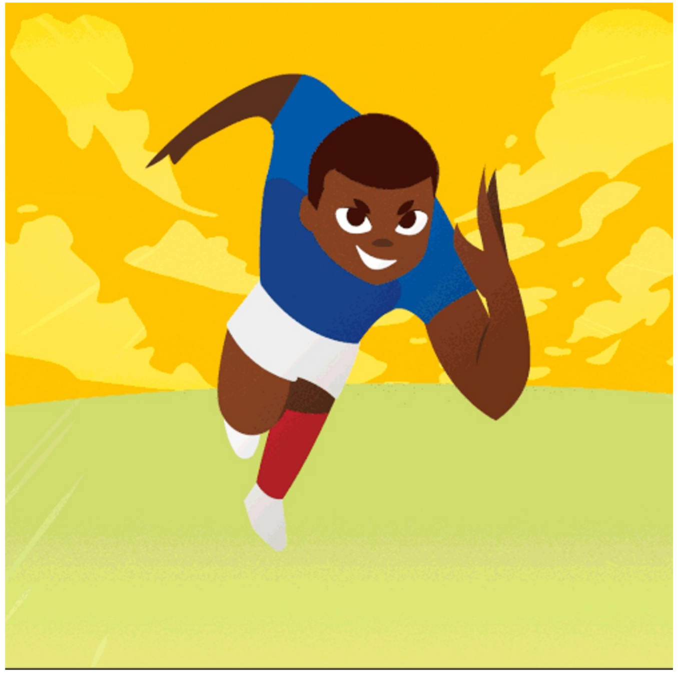

Lively project

Finanziato dall'Unione europea NextGenerationEU
Ministero dell'Università e della Ricerca
Italiadomani
onfoods

LECTURE 2 . MICRONUTRIENTS

Lively project

Vitamins are organic compounds that must be introduced through the diet because the body is unable to produce them in sufficient quantities for the body's needs. Their deficiency causes life-threatening diseases (hence the term vitamin = amine necessary for life).

In relation to their molecular characteristics, they are distinguished into two major categories: those vitamins that are fat-soluble, that is, those that dissolve in fat (A, D, E, K) andThe water-soluble vitamins, that is, those that dissolve in water (B vitamins, C).

VITAMINS	MAIN FUNCTIONS	FOOD SOURCES
**VITAMINS A**	It intervenes in the mechanism of vision. It is necessary for maintaining the integrity of the cornea, skin, mucous membranes, and cell membranes. It intervenes in the synthesis of some hormones and regulation of the immune system..	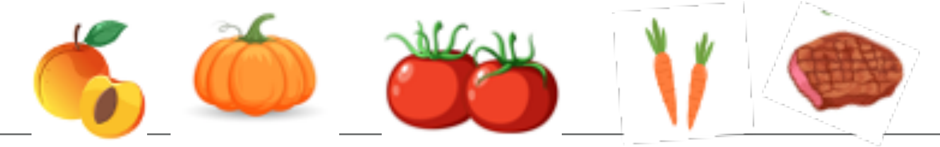
**VITAMINS D**	Intervenes in bone mineralization. Improves calcium absorption.	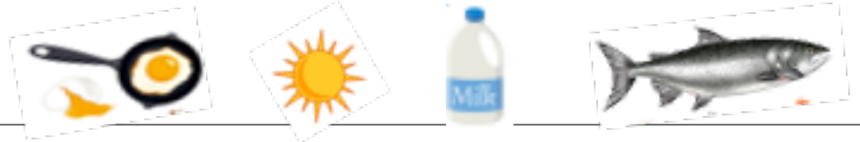
**VITAMINS E**	Antioxidant activity and maintenance of membrane integrity.	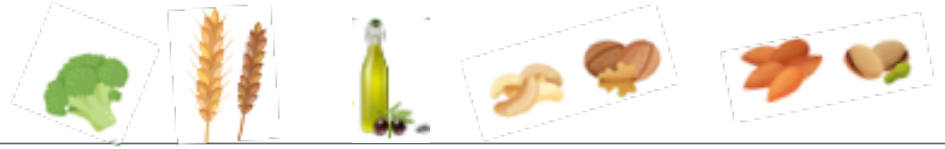
**VITAMINS K**	It intervenes in the synthesis of proteins, which are involved in blood clotting.	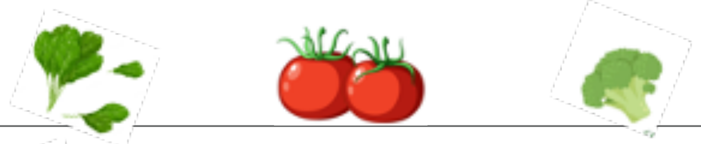
**VITAMINS B12**	Involved in the formation of healthy red blood cells, important for controlling central nervous system metabolism	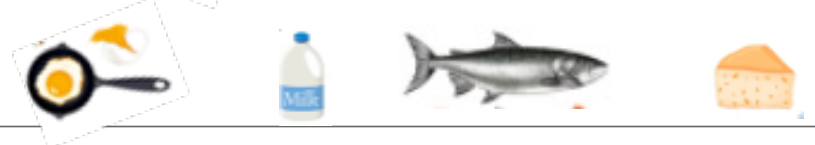
**VITAMINS C**	It has̀ antioxidant activity. Intervenes in collagen synthesis and facilitates iron absorption. It improves respiratory function and wound healing.	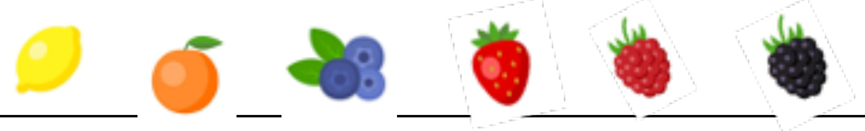

LECTURE 2 . MICRONUTRIENTS

Mineral are divided into major elements (whose requirements are on the order of a gram or a little less) and trace elements, whose requirements are much less (milligrams or micrograms). Each element performs specific functions in our bodies: plastic functions, such as calcium and phosphorus, constituents of bones and teeth, iron a component of the hemoglobin of red blood cells; regulatory functions for example calcium, which regulates muscle contraction and blood clotting, or sodium and potassium, which are involved in the transmission of nerve impulses and the regulation of heart rhythm.

MINERALS	MAIN FUNCTIONS	FOOD SOURCES
**Calcium** (Ca)	Along with phosphorus, it is the major mineral in bones and teeth. It is involved in muscle contraction and relaxation, nerve impulse transmission, blood clotting, blood pressure, and immune defense.	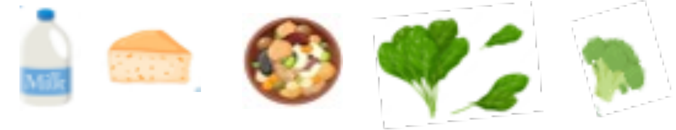
**Phosohorous** (P)	Along with calcium, it is the main mineral in bones and teeth and plays a key role in converting food into energy.	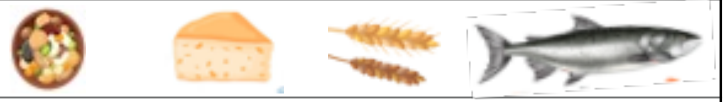
**Sodium** (Na)	It is the main electrolyte of extracellular fluids and is important in nerve impulse transmission.	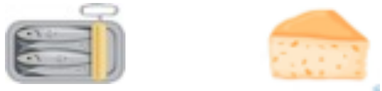
**Potassium** (K)	Electrolyte important for maintaining water balance, nerve impulse transmission, and muscle contraction.	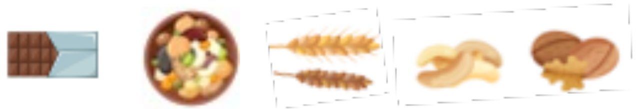
**Iron** (Fe)	A component of hemoglobin and myoglobin, it serves for the transport of oxygen in the blood and muscles.	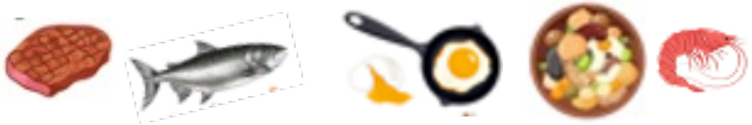
**Iodium** (I)	Constituent of thyroid hormones.	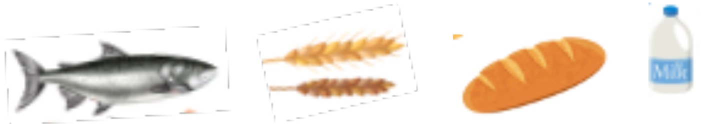

MuLtidimensional school-based and family Involved interVentions, to promote a hEalthy and sustainable LifestYle for the childhood obesity primary prevention

LET’S PLAY!

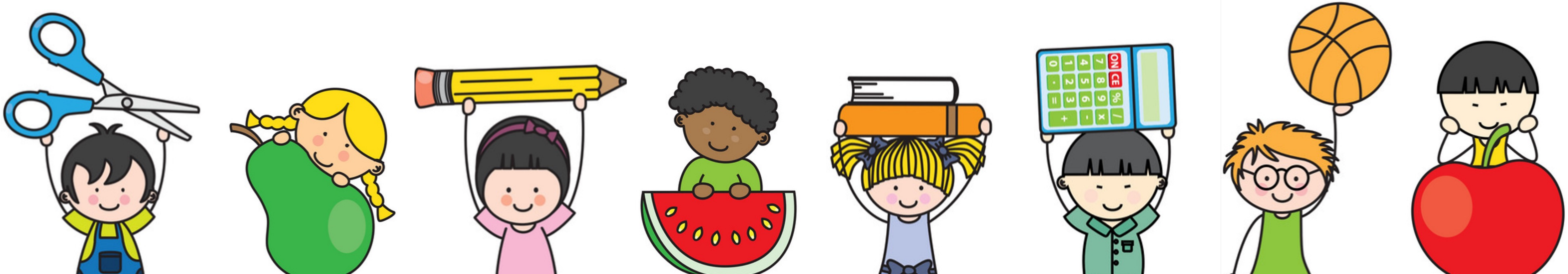

Supplement: Multimedia Appendix 2 [file resprot_v13i1e57509_app2.pdf]
