# Supplementary material for: Multidimensional School-Based and Family-Involved Interventions to Promote a Healthy and Sustainable Lifestyle (LIVELY) for Childhood Obesity Prevention: Study Protocol
Source: JMIR Res Protoc. 2024 Oct 30;13:e57509. doi: 10.2196/57509 (PMC11561434; doi:10.2196/57509)
Supplement: Multimedia Appendix 3 [file resprot_v13i1e57509_app3.docx]

**Appendix 2**

**Figure 1.** **Humanized carrot drawn by a fourth-grade class.**


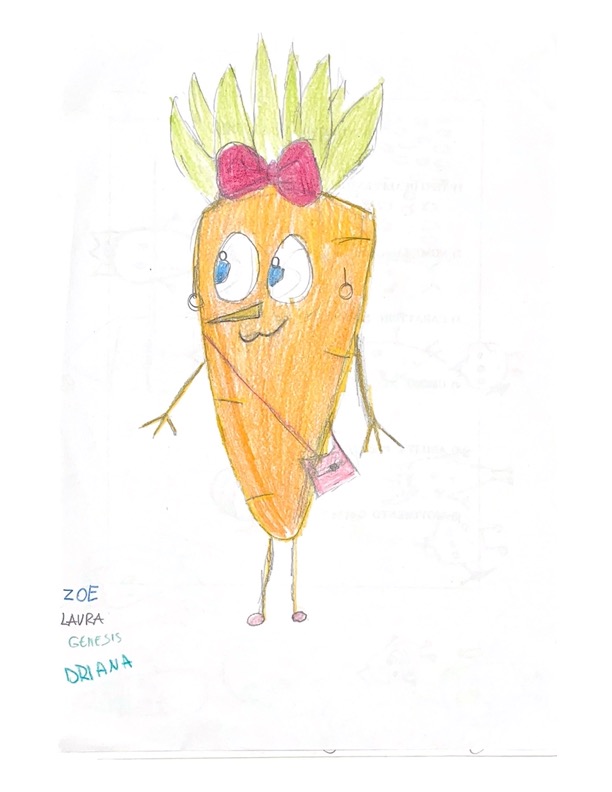


*Figure 1 shows an example of draw pictured by a group of fourth-grade children during the multimedia lab.*

**Figure 2.** **Features of the humanized carrot drawn**


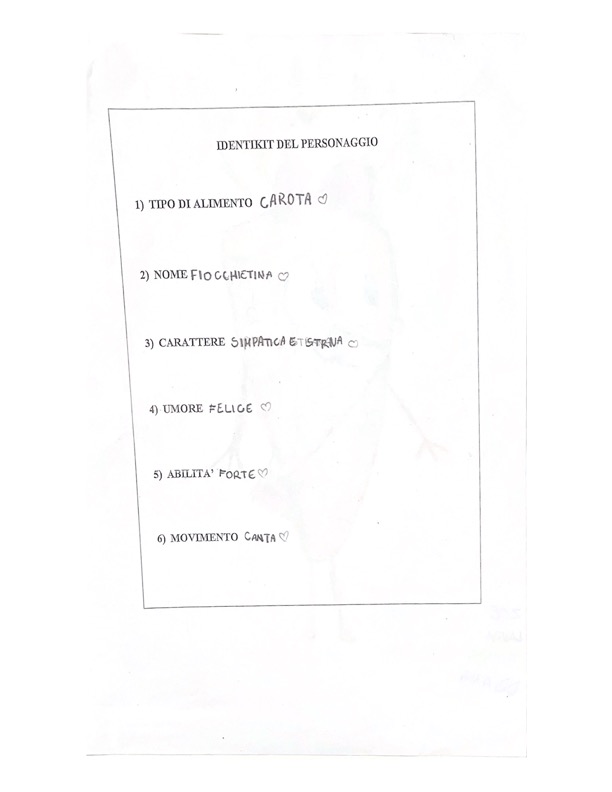


What is it doing: singing

Special power: strenght

Mood: happy

Personality: nice and weird

First name: “Fiocchetina”

Kind of food: Carrot

**Character description**

*Figure 2 shows the requested features of the humanized carrot to define the character*
