# Supplementary material for: Multidimensional School-Based and Family-Involved Interventions to Promote a Healthy and Sustainable Lifestyle (LIVELY) for Childhood Obesity Prevention: Study Protocol
Source: JMIR Res Protoc. 2024 Oct 30;13:e57509. doi: 10.2196/57509 (PMC11561434; doi:10.2196/57509)
Supplement: Multimedia Appendix 4 [file resprot_v13i1e57509_app4.pdf]

SPIRIT

STANDARD PROTOCOL ITEMS: RECOMMENDATIONS FOR INTERVENTIONAL TRIALS

SPIRIT 2013 Checklist: Recommended items to address in a clinical trial protocol and related documents*

Section/item	ItemNo	Description
**Administrative information**		
Title	1	LIVELY: MuLtidimensional school-based and family Involved interVentions, to promote a hEalthy and sustainable LifestYle for the childhood obesity primary prevention, a Study Protocol
Trial registration	2a	ClinicalTrials.gov NCT05966051
	2b	NA
Protocol version	3	Revision 1
Funding	4	Project funded under the National Recovery and Resilience Plan (NRRP), Mission 4 Component 2 Investment 1.3 - Call for proposals No. 341 of 15 March 2022 of Italian Ministry of University and Research funded by the European Union – NextGenerationEU.
Roles and responsibilities	5a	Sara Basilico[1], Maria Vittoria Conti[1], Ilaria Ardoino[2], Chiara Breda[1], Federica Loperfido[1], Elena Klaic[1], Linda Spialtini[1], Andreana Foresta[3], Francesca Orsini[2], Luisa Ojeda Fernandez[3], Stefano Conca Bonizzoni[4], Elisabetta Modena[4], Yasamin Tootoonchi Hamedani[4], Federica Villa[4], Hellas Cena[1,5], Marta Baviera[3], Carlotta Franchi[2,6]<br><br>1. Laboratory of Dietetics and Clinical Nutrition, Department of Public Health, Experimental and Forensic Medicine, University of Pavia, Pavia, Italy<br>2. Laboratory of Pharmacoepidemiology and Human Nutrition, Department of Health Policy, Istituto di Ricerche Farmacologiche Mario Negri IRCCS Milan, Italy<br>3. Laboratory of Cardiovascular Prevention, Department of Health Policy, Istituto di Ricerche Farmacologiche Mario Negri IRCCS, Milan, Italy<br>4. Department of Humanities, University of Pavia, Pavia, Italy<br>5. Clinical nutrition Unit, General Medicine, ICS MAUGERI IRCCS, Pavia, Italy<br>6. Italian Institute for Planetary Health, Milan, Italy
	5b	NA
	5c	The founder does not have a role in the study
	5d	NA
**Introduction**		

Background and rationale	6a	Childhood obesity has become a significant public health concern over the past two decades, posing multifactorial challenges that include modifiable factors like dietary habits and physical activity. Prevention efforts require a comprehensive approach, including educational interventions, collaboration among multidisciplinary teams, and community engagement. Given that schools play a central role in children's lives, they are an ideal setting for promoting healthy habits.
	6b	For this pilot study no comparators are included
Objectives	7	The LIVELY study focuses on assessing the prevalence of overweight and obesity in primary school children and identifying contributing factors within families. Additionally, it aims to implement and evaluate a multidimensional, multidisciplinary intervention to foster a sustainable and healthy lifestyle, ultimately working towards preventing obesity in school-aged children.
Trial design	8	The study is a single arm pre -post study (without control).

Methods: Participants, interventions, and outcomes

Study setting	9	The study setting is a public primary school in the North-West of Milan, Italy.
Eligibility criteria	10	Inclusion criteria:<br>• Age: ≥5 to ≤12 years old<br>• Sex: male and female<br>• Ethnicity: any<br>• School: Istituto Comprensivo "Luigi Cadorna”<br>• Privacy form and informed consent: signed by the parents or legal guardians<br><br>Exclusion criteria:<br>• Age: <5 or >12 years old<br>• School: other schools rather than Istituto Comprensivo "Luigi Cadorna”<br>• Privacy form and informed consent: not signed by the parents or legal guardians<br><br>The intervention will be performed by nutritionist biologists and dieticians.

Interventions	11a	During the school year, each class is individually involved in a multidimensional educational intervention that will cover the topics of healthy and sustainable nutrition and lifestyle. All classes will be conducted during regular school hours and included within the teaching of Civic Education. Six lessons from October 2023 up to April 2024 will be conducted on the following topics: macronutrients, micronutrients, the digestive system, food pyramid, healthy eating plate, lifestyle (sleep habits, screen time and physical activity). Each lesson, every four weeks and lasting 2 hours, will be divided into two parts: the first part of about 30-40 minutes of frontal teaching with the support of interactive slides, and the second part of about 80-90 minutes in which playful activities adapted to the target age group of children will be carried out.
	11b	NA
	11c	To improve adherence to the study, all the privacy forms, information consents and questionnaires were translated in Italian, English and Arabic due to the high multiethnicity of the school. After each lesson, a summary of the content was given to the parents in order to engage them also at home.
	11d	NA
Outcomes	12	The study's primary objectives include:<br>• To investigate prevalence of overweight and obesity among primary school children and its determinants related to children (clinical history, lifestyle habits) and their families (socio-economic status, environmental influences, and behavioral determinants).<br>• to set up and to evaluate the feasibility of a multidimensional school-based educational intervention promoting a healthy and sustainable lifestyle and therefore aim at preventing childhood obesity.<br>• the assessment of possible changes in children's diet and lifestyle, as well as in family behavioral attitudes, at 6 and 12 months from the execution of the interventions.
Participant timeline	13	See Figure 1.
Sample size	14	For this kind of study, it is not needed a sample size calculation, but we expected to enroll about 300/350 children aged 6/12 years within 15/20 classes (in primary school and in the first class of first grade secondary school) based on the availability/willingness of teachers to participate and of the resources.<br>This will allow us to estimate a prevalence of overweight and obesity of about 23-24% with a 20% relative precision and within a 95% confidence interval. This data is in line with those observed in the Okkio alla Salute 2019 survey in the Lombardy region

Recruitment	15	The multidisciplinary team organized an initial meeting at the participating school to introduce the project to the dean and teachers. Teacher representatives took charge of presenting the project to the families of the various classes. Interested families were provided with a study information sheet which was translated in different languages (Arabic and English) due to the high multiethnicity, especially Arabian, of the school.

Methods: Assignment of interventions (for controlled trials)

Allocation:

Sequence generation	16a	NA
Allocation concealment mechanism	16b	NA
Implementation	16c	NA
Blinding (masking)	17a	NA
	17b	NA

Methods: Data collection, management, and analysis

Data collection methods	18a	Data collection will be assessed at three time points: at the base line (T0), at six months from the baseline (T1) and at 12 months from the baseline (T2).<br>• Anthropometric measures (weight, height, waist circumference and bicep circumference): Instruments = digital scale, portable altimeter and flexible centimetre. When: T0, T1 and T2.<br>• Sociodemographic information related to the parents: Instruments = questionnaires. When: T0, T1 and T2.<br>• Sociodemographic information related to the children: Instruments = questionnaires. When: T0, T1 and T2.<br>• Diet and Lifestyle related to the children: Instruments = questionnaires. When: T0, T1 and T2.<br>• Project evaluation: Instruments = questionnaires. When: T1.
	18b	To promote participant retention and complete follow-up questionnaires were translated in Italian, English and Arabic due to the high multiethnicity of the school.
Data management	19	All data will be anonymized and storedby the sponsor.
Statistical methods	20a	Statistical methods for analysing primary and secondary outcomes. Reference to where other details of the statistical analysis plan can be found, if not in the protocol
	20b	Methods for any additional analyses (eg, subgroup and adjusted analyses)

	20c	Definition of analysis population relating to protocol non-adherence (eg, as randomised analysis), and any statistical methods to handle missing data (eg, multiple imputation)
**Methods: Monitoring**		
Data monitoring	21a	This study test an educational intervention thus Data Monitoring Committee (DMC) is not needed.
	21b	NA
Harms	22	NA
Auditing	23	NA
**Ethics and dissemination**		
Research ethics approval	24	Fondazione IRCCS Istituto Neurologico Carlo Besta Ethics Committee (protocol 11) - January 2023.
Protocol amendments	25	NA
Consent or assent	26a	The informed consents from parents or legal guardians of the children will be collected by the investigators.
	26b	NA
Confidentiality	27	All data will be anonymized and storedby the sponsor.
Declaration of interests	28	None declared
Access to data	29	The final trial dataset will be accessed only to authorised personal by the sponsor by investigators.
Ancillary and post-trial care	30	NA
Dissemination policy	31a	All results in anonymized form and materials produced during the study will be disclosed while respecting the privacy of participants.
	31b	Authorship eligibility guidelines and any intended use of professional writers
	31c	The full protocol and participant-level dataset will be available upon request to the corresponding author.
**Appendices**		
Informed consent materials	32	Model consent form and other related documentation given to participants and authorised surrogates
Biological specimens	33	NA
